# Ericoid mycorrhizal growth response is influenced by host plant phylogeny

**DOI:** 10.1007/s00572-025-01223-6

**Published:** 2025-08-13

**Authors:** Alice S. Neves, Laura G. van Galen, Martin Vohník, Martina Peter, Elena Martino, Thomas W. Crowther, Camille S. Delavaux

**Affiliations:** 1 Department of Environmental Systems Science, Institute of Integrative Biology, ETH Zürich (Swiss Federal Institute of Technology), Zürich, 8092 Switzerland; 2 Department of Mycorrhizal Symbioses, Institute of Botany, Czech Academy of Sciences, Průhonice, 25243 Czechia; 3 Biodiversity and Conservation Biology, Swiss Federal Institute for Forest, Snow and Landscape Research WSL, Zuercherstrasse 111, Birmensdorf, 8903 Switzerland; 4 Department of Life Sciences and Systems Biology, University of Torino, V. le Mattioli 25, Torino, 10125 Italy; 5 Université de Lorraine, INRAE, UMR Interactions Arbres/Microorganismes, Centre INRAE Grand Est Nancy, Champenoux, France

**Keywords:** Ericoid mycorrhizal fungi, Ericaceae, Mycorrhizal growth response, Heathland, Wetland

## Abstract

Ericoid mycorrhizal (ErM) fungi (ErMF) are crucial for the establishment of thousands of ericaceous species in heathlands and wetlands by increasing their tolerance to harsh conditions and improving nutrient uptake. However, ErM research has largely focused on a limited number of host species and four ErMF species (especially *Hyaloscypha hepaticicola* and *Oidiodendron maius*, to a lesser extent *H. bicolor*/*H. finlandica* and *H. variabilis*). Therefore, the degree to which other ericaceous plants and ErMF form functional associations, and corresponding benefits for plant growth, are not well understood. As such, we lack a clear understanding of how changes in fungal partners may influence plant fitness. To address this gap, we conducted a greenhouse experiment with nine ericaceous plant species and eight ErMF isolates to expand baseline knowledge regarding the effects of the ErM symbiosis on host plant growth. By analyzing ErM root colonization and host plant growth response, we observed that the mycorrhizal growth response (MGR) was variable and depended on plant and fungal identity. Moreover, overall inoculation effects on plant growth were independent from colonization levels. Finally, we found evidence that MGR was influenced by plant phylogeny. These results expand our basic understanding of the ErM symbiosis and provide valuable information for future restoration and conservation efforts.

**Supplementary Information:**

The online version contains supplementary material available at 10.1007/s00572-025-01223-6.

## Introduction

Although the ericoid mycorrhizal (ErM) symbiosis has been historically understudied compared to the other main mycorrhizal types (i.e., arbuscular (AM) and orchid endomycorrhizas and ectomycorrhizas (EcM), it represents the dominant mycorrhizal strategy in heathlands and wetland ecosystems (Read et al. 2004; Smith and Read 2008). The ErM symbiosis involves ErM fungi (ErMF) and members of the early anther inversion clade of Ericaceae (i.e., the core Ericaceae, referred to as ericaceous plants in the following text). This symbiosis is characterized by compact intracellular hyphal coils involved in nutrient exchange, which are formed in the rhizodermal cells of the hosts’ delicate hair roots (Leopold 2016; Vohník 2020; Grelet et al. 2016). ErMF are crucial to ericaceous plant establishment and survival in their environment, which often contain waterlogged and acidic soils, rich in recalcitrant organic compounds and poor in mineral nutrients (Cairney and Meharg 2003; Read 1991; Read and Kerley 1999). In these harsh habitats, ErMF provide their host plants with improved nutrient uptake, predominantly inorganic nitrogen and phosphorus, thanks to their rich hydrolytic enzyme arsenal enabling them to decompose complex soil organic matter (SOM), allowing host plants to access otherwise unavailable organic nutrients (Martino et al. 2018; Perotto et al. 2002, 2012).

It is noteworthy that wetland (i.e. ecosystems in the edge between aquatic and terrestrial ecosystems; Kingsford et al. 2016) and heathland (i.e. shrublands characterized by low concentration of soil nutrients; Fagúndez 2013) habitats in which the ErM symbiosis occurs provide valuable ecosystem services, including protection of a unique biodiversity, climate regulation, and elevation of soil carbon stocks (Fagúndez 2013; Kingsford et al. 2016). Nevertheless, a large proportion of these sites are degraded or threatened, and so there is a critical need to develop effective restoration methods (Fagúndez 2013; Hartley et al. 2021; Kingsford et al. 2016; Mitsch et al. 2013; Mitsch and Gosselink 2000; Webb 1998; Zedler and Kercher 2005). Consequently, a thorough understanding of the functioning of the ErM symbiosis in these systems is needed to improve conservation and restoration and the essential services these ecosystems provide. However, until now, research on the ErM symbiosis has been restricted in the breadth of plant and fungal species studied, limiting our understanding of how plant and fungal identities influence this symbiosis.

Despite the ErM core Ericaceae encompassing five subfamilies (Vohník 2020), recent research has mostly only focused on economically valuable genera such as *Vaccinium* and *Rhododendron* (e.g., Ważny et al. 2022; Wei et al. 2022). Similarly, research has also been limited to a small number of ErMF, with most efforts focused on the four archetypal ErMF species (especially *Hyaloscypha hepaticicola* and *Oidiodendron maius*, to a lesser extent *H. bicolor*/*H. finlandica* and *H. variabilis*) (Straker 1996; Leopold 2016; Vohník 2020). This leaves considerable gaps in our understanding of the formation and function of the ErM symbiosis. For example, of particular interest to both agriculture and restoration are the growth benefits obtained from the symbiosis, measured as the mycorrhizal growth response (MGR) (Köhl et al. 2016). Therefore, baseline information regarding the compatibility and resulting benefits of the ErM symbiosis across a wider range of ericaceous plants and ErMF will serve as a first step to address these knowledge gaps.

An important open question is whether ericaceous plants and ErMF associate with and respond to all potential symbiotic partners (i.e., they are generalists) or if the association and/or response is more selective (i.e., they exhibit high specificity; Clayton et al. 2004). Specificity can be assessed as association specificity, describing the range of pairings that lead to colonization and the formation of mycorrhizas, or response specificity, describing the range of pairings that lead to growth benefit (Hart and Klironomos 2003). Previous work in AM and EcM systems has shown that association specificity varies across mycorrhizal types, with EcM systems generally exhibiting higher specificity than AM systems (Carriconde et al. 2019; Lofgren et al. 2018; Tedersoo et al. 2008; van Galen et al. 2023). However, response specificity has been shown to vary within both AM and EcM, with the identity of both partners strongly impacting growth response (Hoeksema et al. 2018). At present, we lack a basic understanding of both association and response specificity in the ErM system. Some work does show evidence for both high and low association specificity, but no robust assessment exists for response specificity (Perotto et al. 2002; Põlme et al. 2018; Van Geel et al. 2020). Ultimately, examining a wider range of plant and fungal pairings is needed to better test specificity in the ERM symbiosis.

Phylogenetic relatedness may be an important driver of plant response specificity in ErM systems. Phylogeny has been shown to be useful in predicting plant growth response to mycorrhizal fungi (Hoeksema et al. 2018; Reinhart et al. 2012). Recently, it has been shown that evolutionary history of both plant hosts and fungal symbionts plays a major role in MGR across both AM and EcM plant species (Hoeksema et al. 2018). However, Reinhart et al. (2012) reported that functional traits are also useful (i.e., life history, photosynthetic pathway, root to shoot ratios, etc.). If there is a strong and predictable phylogenetic signal in growth response across ericaceous plants, this would have important implications for use of ErMF in conservation. Instead of needing to conduct experiments across hundreds or thousands of plant species, researchers could target gaps in the phylogeny to generate a predictive phylogenetic framework of ErM responsiveness.

Here, we use a greenhouse experiment with nine ericaceous plant species and eight ErMF isolates grown together in 72 unique plant-fungal combinations to test the effect of the ErM symbiosis on plant growth. By expanding the number of plants and fungal species in comparison to those previously studied, this experiment aims to (1) provide a greater understanding of the benefits received by the plants from unique pairings within different ErMF, (2) examine the levels of association and response specificities shown by different ericaceous plants and ErMF, and (3) test whether the observed ErM colonization and benefits are influenced by plant phylogenetic history.

## Materials and methods

### Plant and fungal material

We conducted a greenhouse experiment using nine ericaceous species (Table 1A) and eight ErMF isolates (from seven ErMF species; Table 1B) from wetland and heathland systems. The plant species were native to Europe, North America, and Asia (Table 1A). Seeds were purchased from Semences du Puy (www.semencesdupuy.com) and were sowed in autoclaved soil (1:1 vermiculite and white peat) and grown for 3 months in a greenhouse at 20 °C before experimental set up. *Vaccinium angustifolium* required cold stratification to break dormancy; therefore, its seeds were stored in Petri dishes with moist paper towel at 4 ºC for four weeks prior to sowing. The seedlings were watered every Monday, Wednesday, and Friday to ensure consistent adequate moisture.

Eight ErMF isolates (Table 1B) were isolated from ericaceous plants in Europe and were obtained from the Swiss Federal Institute for Forest, Snow and Landscape Research WSL and the Institute of Botany, Czech Academy of Sciences (CAS). Cultures were maintained on Modified Leonian’s agar (MLA) (Malloch 1981) at 18 ºC. All of the ErMF species used have been previously confirmed to form ErM (Vohník 2020; Vohník et al. 2012, 2016). The fungal isolate pool is composed by ErMF isolates that have been more heavily studied (*H. hepaticicola*,* O. maius*, and *H. variabilis*) but also by isolates that have received less attention (*H. bicolor*,* H. gryndleri*,* K. argillacea*, and *Serendipitaceae sp.*). Sanger sequences of the strains *H. hepaticicola* PK 135-3, *Oidiodendron maius* JPK 11, and *Kurtia* (*Ku.*) *argillacea* JPK 87 were obtained (see Supplementary material 1 for detailed sequencing protocol).

**Table 1:** Studied species A) native range of the plant species used in this study. Source: plants of the word online, Royal Botanic Gardens, Kew (POWO 2024). B) ErMF isolates used in this study. CBS = Westerdijk Fungal Biodiversity Institute (formerly centraalbureau voor Schimmelcultures), Utrecht, Netherlands; ITS = ITS1-5.8S-ITS2 ribosomal RNA gene; LSU = 28S ribosomal RNA gene; NA = not available; NCBI = National Center for Biotechnology Information, USA; UAMH = University of Alberta Microfungal Herbarium (https://www.uamh.ca/); ICMP = International Collection of Microorganisms from plants (https://www.landcareresearch.co.nz/tools-and-resources/collections/icmp-culture-collection/; https://scd.landcareresearch.co.nz/)

A				
Plant species		Native range		
*Calluna vulgaris*		Macaronesia, Europe to Central Siberia, Northern Morocco		
*Gaultheria shallon*		Alaska to Western USA		
*Kalmia latifolia*		Eastern USA		
*Pieris japonica*		South-eastern China (to Hubei), Central and Southern Japan, Taiwan		
*Rhododendron arboreum*		Indian Subcontinent to Southern Tibet and Northern Myanmar		
*Rhododendron ferrugineum*		Pyrenees, Eastern France to North-western Balkan Peninsula, Southern Poland (Karkonosze Mountains)		
*Vaccinium angustifolium*		Central and Eastern Canada to Northern, Central and Eastern USA		
*Vaccinium myrtillus*		Greenland, Temperate Eurasia, Western Canada to North-western and Central Western USA		
*Vaccinium vitis-idaea*		Subarctic and Temperate Northern Hemisphere		
B				
Fungal species	Isolate (host and country of origin)		Reference sequence in GenBank at NCBI	Reference
*Hyaloscypha bicolor*	UAMH 11,274/ICMP 18,549 (*V. vitis-idaea*, Scotland)		PRJNA196026/LXPI00000000	(Grelet et al. 2009)
*Hyaloscypha gryndleri*	MGR-3/CBS 145,338 (*V. myrtillus*, Czechia)		MZ520786 (ITS) MZ520775 (LSU)	( Vohník et al. 2022 )
*Hyaloscypha hepaticicola*	PK 135-3 (*C. vulgaris*, Czechia)		PV164618 (ITS)	this study
*Hyaloscypha hepaticicola*	UAMH 7357/ICMP 18,553 (*C. vulgaris*, England)		PRJNA263050/LYBP00000000	( Read 1974 )
*Hyaloscypha variabilis*	UAMH 11,265/ICMP 18,552 (*V. vitis-idaea*, Scotland)		PRJNA200595/LXPR00000000	( Grelet et al. 2009 )
*Kurtia argillacea*	JPK 87 (*V*. *myrtillus*, Norway)		PV164617 (ITS) HE802996 (LSU)	( Vohník et al. 2012 ), this study
*Oidiodendron maius*	JPK 11 (*V. myrtillus*, Norway)		PV164619 (ITS)	this study
*Serendipitaceae* sp.	JPK 132 (*Vaccinium* sp., Norway)		OP863025 (ITS) KT762614 (LSU)	( Vohník et al. 2016; Vohník and Réblová 2023 )

### Experimental design

The experiment consisted of individuals from each plant species grown in combination with one of the fungal species or as non-inoculated controls (deionized (DI) water). Therefore, there were 72 unique plant–fungus treatment combinations and 9 non-inoculated controls. Treatments involving *Calluna vulgaris*, *Rhododendron arboreum*, *R. ferrugineum*, *Vaccinium angustifolium*, and *V. myrtillus* were replicated 10 times. Treatments with the remaining plant species were only replicated seven times due to lower germination rates. All non-inoculated controls had 10 replicates. Seedlings were grown in plastic pots that were 4 cm in diameter and had a volume of 100 mL. We used a 1:1 mix of white peat and vermiculite as growing substrate to reproduce as closely as possible the highly acidic nutrient-poor soil characterizing the ericaceous plants in their native ranges.

#### Fungal cultures

Fungal cultures were prepared by applying two methods to maximize the chance to get a successful growth: (a) MLA solid plates (9 cm diameter) inoculated with each strain were placed in an incubator at 18 ºC, and (b) 400 ml of MLA liquid cultures for each strain were prepared in glass bottles, placed on a shaker incubator (120 rpm), and kept at room temperature for 4 weeks. To prepare the fungal inoculum for plant inoculation, for each fungal strain the mycelium from MLA liquid cultures was mixed with the corresponding mycelium carefully scraped from five agar plates and 150 mL of DI water were added. For *Ku. argillacea* JPK 87 only MLA liquid cultures were used to prepare the fungal inoculum as MLA solid cultures were not available. Each individual fungal inoculum was blended for 1 s three times and stored at 4 ºC until plant inoculation (maximum 7 days). The blender was sterilized in a 10% bleach solution for 15 min and thoroughly rinsed with running water between each isolate.

#### Pot and soil preparation

The trays and pots used in the experiment were washed with soap and water, surface-sterilized in a 10% bleach solution for 15 min, and rinsed with running water to ensure maximum cleanliness. To prepare the growth substrate, white peat was soaked overnight in a 1:10 solution of wetting agent and water. Vermiculite was added to the peat in a 1:1 ratio and the mixture was autoclaved twice, at 120 ºC, with a 24 h interval between each autoclave cycle (Vohník 2020).

#### Planting and harvest

As each seedling was planted, 2 mL of the fungal inoculum previously prepared were added directly on the roots using a sterile plastic pipette. Extreme care was taken to minimize contamination during this phase by regularly sterilizing all equipment used and the potting area. The pots were randomly positioned in blocks in the greenhouse to account for any possible spatial variation, with pots spaced at least 2 cm apart to avoid any contamination between treatments. To ensure that no runoff from one pot would have contact with another, flipped trays were placed below the tray supporting the pots. The shoot height was measured one week after planting to provide the initial height of each seedling to account for any differences in initial heights. At this stage, we also replaced dead seedlings with new ones. The seedlings were hand-watered with tap water using a small bottle to ensure no water splashed between pots every Monday, Wednesday, and Friday for four months until harvested. During harvest, plant shoots were cut at the soil level. Roots were gently cleaned in tap water to remove the adhering soil and stored in 1.5 mL Eppendorf tubes with 50% ethanol at 4 ºC for the root colonization assessment.

### Root colonization assessment

To verify whether the fungi and plant roots had formed the ErM symbiosis, we performed a root colonization assessment for three replicates of each unique combination (239 in total; four combinations only contained two replicates due to processing issues). A representative subsample of 10 root pieces (1–2 cm in length) was taken from each seedling and placed in a histological cassette. The subsamples were stained using a modified protocol from both Vohník (2020) and the International Collection of (Vesicular) Arbuscular Mycorrhizal Fungi (INVAM 2024a). The roots were cleared by autoclaving at 121 ºC for 20 min in a 10% KOH solution, then washed with tap water 10 times, and acidified in a 2% HCl solution at room temperature for 30 min. The samples were stained with a 0.05% solution of trypan blue in lactoglycerol (1:1:1 glycerol, lactic acid, DI water) overnight at room temperature. Finally, roots were washed with tap water 10 times and de-stained in lactoglycerol at 4 ºC for 4 days. The samples were mounted in microscope slides with Polyvinyl-Lacto-Glycerol (INVAM 2024b).

**Fig. 1:**
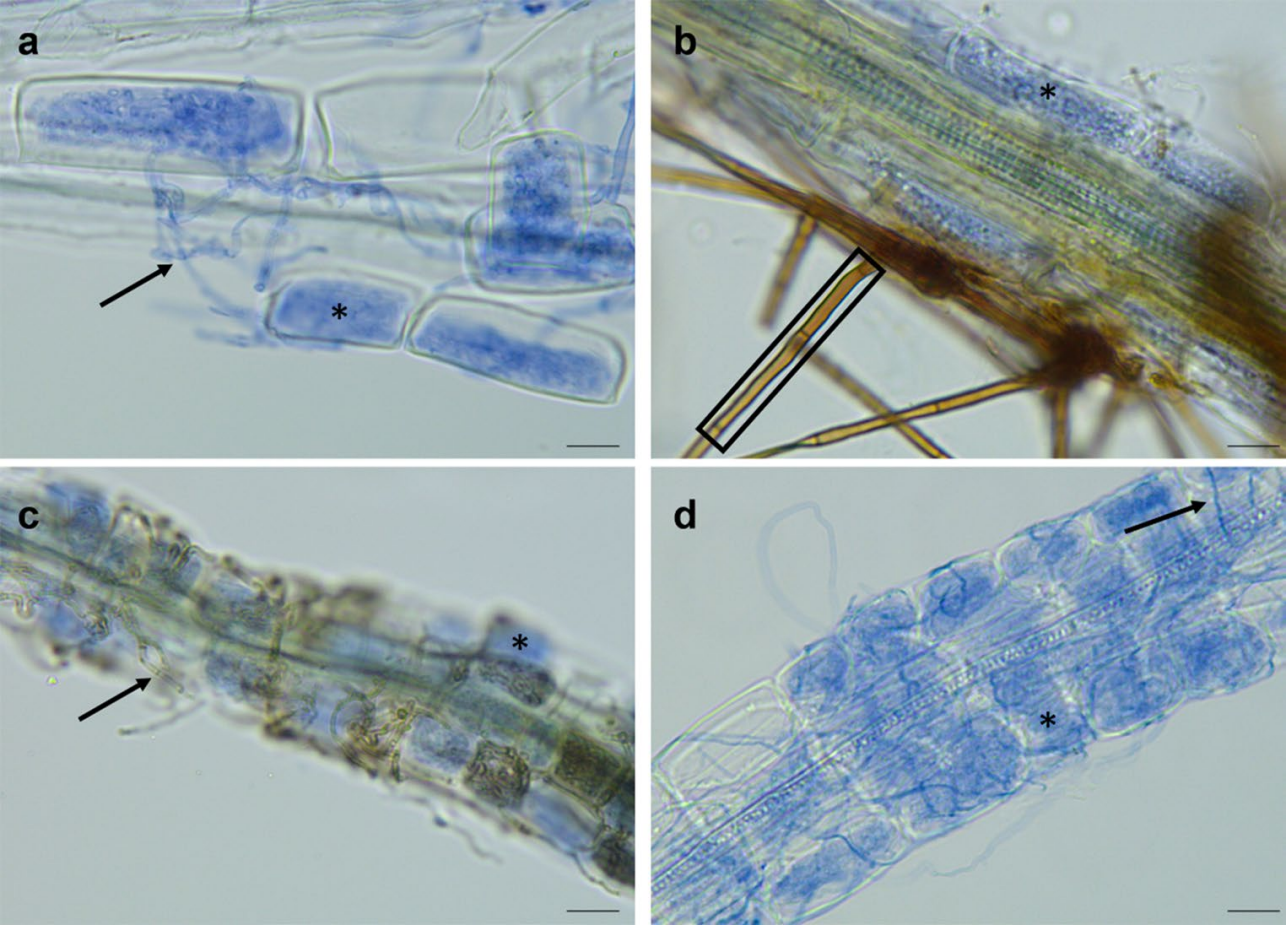
Examples of ericoid mycorrhizal structures observed in the experiment. Ericoid mycorrhizal fungal colonization is characterized by the presence of intracellular hyphal coils inside the host’s rhizodermis (asterisk) and surface hyphae (arrow), as well as the presence of conidiophores in the case of *Oidiodendron maius* (rectangle). Fungal tissue is stained blue. **a** *Rhododendron ferrugineum* - *Kurtia argillacea* JPK 87; **b** *Gaultheria shallon* - *Oidiodendron maius* JPK 11; **c** *Vaccinium vitis-idaea* - *H. hepaticicola* PK 135-3; **d** *Kalmia latifolia*– *Serendipitaceae* sp. JPK 132. Scale bars are 60 μm

We evaluated the colonization rate of each sample by examining intracellular hyphal coils, hyphae, and conidiophores using the magnified intersections method (400x magnification, McGonigle et al. 1990). The presence of fine hyphal coils in the rhizodermal cells is the feature that best relates to ErMF colonization, but surface hyphae also indicate fungal presence (Fig. 1). Additionally, conidiophores are asexual reproductive structures that are formed in some species, such as *Odiodendron* spp. (Barron 1962). The magnified intersection method involved assessing vertical transects along each slide that intersected with the root segments. Each intersection was scored for the presence or absence of coils, hyphae, and conidiophores. The number of root intersections per replicate ranged from 50 to 449, except for four replicates that only had 37, 44, 45, and 46 root intersections due to the small size of the roots.

### Plant response measurements

We measured plant biomass to assess plant response to the different fungal treatments. Aboveground biomass was measured by weighing the harvested shoots after drying at 60 ºC for at least one week. Measuring belowground biomass required first calculating the wet:dry weight ratio so that the dry weight of the subsample used in the colonization assessment could be approximated for those seedlings. Therefore, we first measured the wet weight of the total root system for all seedlings and re-measured the wet weight of the 239 seedlings used in the colonization assessment after subsampling. This allowed us to estimate the wet weight of the subsample (total root sample weight minus the remaining root sample weight). We then dried the root systems at 60 ºC for at least one week and re-weighed. We calculated the ratio of wet:dry weight for each seedling (Supplementary Figure S1), used the mean ratio for seedlings within each plant species to approximate the dry weight of the subsample used to assess colonization, and added this back to the dry weight of the rest of the sample. For 87 seedlings, dry weight could not be measured because they were either used in their entirety in the staining protocol (32 samples), were lost during the drying process, or were too small to register on the scale (55 samples weighed less than 0.0001 g). In those cases, the mean wet:dry weight ratio for the relevant plant species was used to approximate their dry weight from the measured wet weight. To evaluate the accuracy of these approximations, we used the ratio to predict the dry weight of all seedlings and compared the predicted dry weight to the actual dry weight. The R^2^ between the actual and predicted values ranged from 0.86 to 0.99 for all the plant species (Supplementary Fig. S1a-b), indicating that this estimation was a good proxy. For *V. myrtillus* one outlier was removed (Supplementary Fig. S1b-c).

### Statistical analysis

Data processing and statistical analysis were performed using R v. 4.3.1 (R Core Team 2023). All plots were generated with the package “ggplot2” v. 3.4.4 (Wickham 2016).

#### Inoculation success

We performed a binomial generalized linear mixed model with the package “glmmTMB” v. 1.1.9 (Brooks et al. 2017) to test whether the proportion of root cells colonized with coils differed between the inoculated and non-inoculated (control) seedlings. We used a two-response binomial variable of colonized to non-colonized intersects, which weights each observation by total root intersects assessed. The model included plant species, fungal treatment, and their interaction as independent variables. We used the “emmeans” package v. 1.10.0 (Lenth 2024) and the “pairs” function from base R to compare differences in the proportion of colonized cells between each unique combination, specifically differences between the control and each fungal treatment within each plant species (i.e., we supplied the “emmeans” function with the plant-fungus interaction term; see Supplementary Table S1). Statistical models could not be performed to evaluate hyphae and conidiophores due to their low presence.

#### Mycorrhizal growth response

MGR was calculated to assess the difference in (dry) biomass between the inoculated and control seedlings for above, belowground and total biomass, therefore there is no value for MGR for the control treatments. This is a common metric used as a proxy for mycorrhizal benefit (Köhl et al. 2016) and is calculated according to Eq. 1. We calculated MGR for 631 seedlings out of the initial 714 seedlings that were planted, because 61 seedlings did not survive during the greenhouse experiment and 22 seedlings were lost during the harvesting and data collection process.

1 $$\:\frac{Biomass\:of\:ErM\:inoculated\:plant\:-\left(Average\:biomass\:of\:non-inoculated\:plants\right)}{\left(Average\:biomass\:of\:non-inoculated\:plants\right)}$$

We fitted the MGR values into a linear mixed-effects model using the package “lme4” v. 1.1.35.1 (Bates et al. 2015) with independent variables of fungal treatment, plant species, and their interaction. We also included the initial height of each plant as a covariate and accounted for spatial non-independence by including the block number (1 to 8) representing the position of seedlings in the greenhouse as a random effect. The “anova” function from package “stats” v. 4.3.1 (R Core Team 2023) was used to evaluate variable significance. Subsequently, the “emmeans” and “pairs” functions were used to conduct pairwise comparisons between all possible levels of the plant and fungal treatments (see Supplementary Tables S2a-c, S3a-c, S4a-c). Additionally, we examined the overall MGR for plants across fungal species (using the “emmeans” package as described above, see Supplementary Tables S2d, S3d, S4d).

We further tested whether MGR could be predicted by the colonization rate using the subset of 239 seedlings for which colonization was assessed. We used a model selection approach with linear mixed-effects models (as described above) to test whether the effect of colonization on MGR was dependent on the plant or fungal species. We fitted (a) a model with the three-way interaction (colonization*fungal species*plant species) and (b) a model with the three possible two-way interactions. No interactions were statistically significant, and so we fitted a final simplified model without interactions. Fungal species, plant species, and initial height were included as covariates, and the block number was included as a random effect (see Supplementary Table S5).

#### Shoot: root biomass allocation

We calculated the difference in the shoot: root dry weight ratio of the inoculated vs. control plants in the same way that we calculated MGR from biomass using Eq. 2.

2 $$\:\frac{Shoot:root\:ratio\:of\:ErM\:inoculated\:plant\:-\left(Average\:shoot:root\:ratio\:of\:non-inoculated\:plants\right)}{\left(Average\:shoot:root\:ratio\:of\:non-inoculated\:plants\right)}$$

We did this to evaluate how plants respond to ErMF presence regarding the allocation of their resources– whether resources are shifted towards the development of the shoot or the roots. The values that we obtained were fitted into a linear mixed-effects model as described for the model testing the effect of plant and fungal treatments on MGR. There was no significant interaction between plant and fungal species, and so the differences in the shoot: root ratio between treatment levels were examined separately for plant and fungal species (using the “emmeans” package as described above, see Supplementary Table S6).

#### Plant phylogenetic signal

We tested if plant phylogenetic relatedness was associated with MGR resulting from different fungal treatments. We did this by calculating the phylogenetic signal metrics Blomberg’s K (Blomberg et al. 2003) and Pagel’s λ (Pagel 1999) using the “phytools” package v. 2.1-1 (Revell 2024). We retrieved the phylogenetic tree of the study plant species from the mega-tree GBOTB (Smith and Brown 2018; Zanne et al. 2014) using the R packages “ape” v. 5.7.1 (Paradis and Schliep 2019) and “pez” v. 1.2.4 (Pearse et al. 2015). We performed the calculations using this tree and the output from the “emmeans” summary (the estimate and standard error) of the model that analyzed the effect of plant-fungal treatment on MGR. We used the model output rather than the raw data to ensure that any variation in MGR due to the covariates and random factors included in the model was accounted for (see Supplementary Table S7). The model estimates of MGR for each plant-fungal combination was plotted as a heat map using the “phytools” package v. 2.1.1 (Revell 2024).

## Results

### Inoculation success

**Fig. 2:**
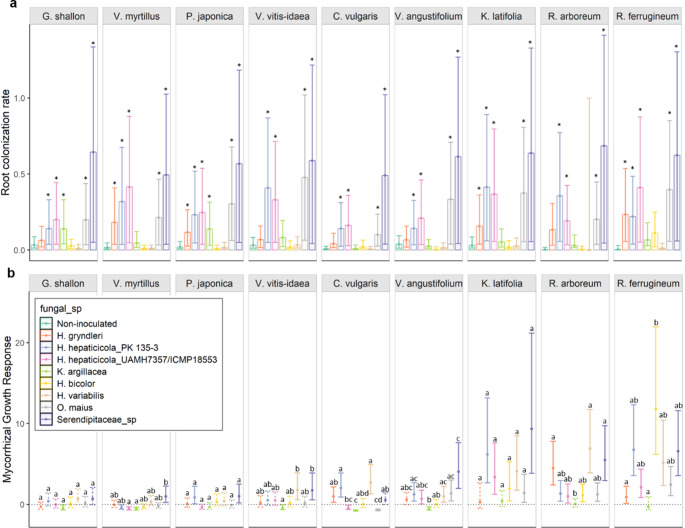
Mycorrhizal growth response depends on plant-fungal combination. Proportion of the rhizodermal cells that contained ErMF hyphal coils (**a**)*.* Within the same plant species, the asterisks indicate a significant difference between the proportion of colonized cells in inoculated and non-inoculated control plants (*p*-value: < 0.05). *n* = 239, three replicates per unique combination were subsampled and stained to be scored for the presence of fungal coils, except for *K. latifolia*–*H. gryndleri* MGR-3, *Calluna vulgaris*–*H. gryndleri* MGR-3, *Pieris japonica*–*Oidiodendron maius* JPK 11, *Gaultheria shallon*–*H. hepaticicola* PK 135-3, and *R. ferrugineum*–*Ku. argillacea* JPK 87 which only have two replicates and *V. angustifolium*–*H. gryndleri* MGR-3 that has four replicates. Mycorrhizal growth response (MGR) for total biomass of the plants studied (**b**)*.* Plots show MGR estimated marginal mean values (circles) and the 95% confidence intervals for each plant-fungus unique combination. Non-significant MGRs (where confidence intervals include zero) are represented by dashed lines. Within the same plant species, significantly different MGRs have different letters. *n* = 631, ranging from 4 to 10 replicates per plant-fungal unique combination, except for *Kalmia latifolia*–*Hyaloscypha gryndleri* MGR-3 and *Rhododendron ferrugineum*–*Kurtia argillacea* JPK 87 which only have 2 replicates

The inoculation with ErMF led to successful colonization in the majority of cases. Specifically, 83% of the inoculated seedlings contained intracellular hyphal coils, 71% surface hyphae, and 12% conidiophores (Fig. 2a). As expected, conidiophores were almost exclusively present in the seedlings inoculated with *O. maius* (Supplementary Fig. S2). Root colonization was also observed in certain control seedlings, indicating some level of fungal spread between treatments or (aerial) contamination. This contamination is expected and common with ErMF, as they can live independently of their host plants (Martino et al. 2018) and the dispersal mechanisms of ErMF are also not currently known (Grelet et al. 2010, 2016). Furthermore, it is less likely that the contamination observed was due to cross-contamination than due to alien airborne contamination. This is because only four of the seven ErMF species have been reported to produce spores. *Hyaloscypha hepaticicola* (Read 1974) and *Ku. argillacea* (Bresadola 1898) produce macroscopic sporocarps with sexual spores not observed in this study. *H. bicolor* and *O. maius* produce asexual spores (conidia), only being observed once in an in vitro agar culture for the first (Fehrer et al. 2019) but regularly for the later (Barron 1962). Conidiophores typical for *O. maius* were observed in this experiment and only in certain contaminated pairings. A high-throughput sequencing approach could have been used to identify exactly which fungi were in each seedling at the end of the study, but would have been difficult due to the size of the experiment and the compatibility of different fungi with different primer sets. Despite this known issue, results from this study still contribute to better understanding ErMF response association, specificity, and growth response.

The presence of fungal colonization in the control seedlings meant that we were unable to reliably quantify association specificity for some plant-fungal combinations. Specifically, plant-fungal combinations where the control did not have significantly lower colonization than treated seedlings could not be meaningfully assessed for association with the fungal inocula of that treatment. However, coil amount in the control seedlings was significantly lower than that observed in the inoculated plants for most plant species, with the exception of those inoculated with *H. variabilis* UAMH 11,265 and *H. bicolor* UAMH 11,274 and in some cases *H. gryndleri* MGR-3 and *Ku. argillacea* JPK 87 (Fig. 2a, Supplementary Table S1). Still, patterns observed for these combinations may be influenced by the contamination from more than one fungal isolate. For MGR however, contamination of controls should only reduce our ability to detect significant results. The presence of fungi in the control treatment might reduce the magnitude of MGR values, potentially producing Type II errors (i.e. false non-significant results). Significant differences are therefore conservative and can still be attributed to fungal benefits.

Across the combinations where treated seedlings show significantly higher colonization than controls, we were able to draw likely patterns of association specificity. We found that for *H. hepaticicola* PK 135-3 and UAMH 7357, *O. maius* JPK 11, and *Serendipitaceae* sp. JPK 132, the percentage of coils formed was significantly higher in the inoculated seedlings compared to control seedlings for all nine plant species, indicating that these fungal isolates are likely association generalists (Fig. 2a). Conversely, for *H. gryndleri* MGR-3, the number of coils formed in the inoculated seedlings was significantly higher than in the non-inoculated ones only for the plant species *V. myrtillus*,* P. japonica*,* K. latifolia*, and *R. ferrugineum*, and the inoculation with *Ku. argillacea* JPK 87 only significantly increased coil formation for *P. japonica* and *G. shallon* (Fig. 2a), possibly indicating higher association specificity for these fungal isolates.

Root coil formation was not related to total MGR (*p*-value = 0.560), aboveground MGR (*p*-value = 0.623), or belowground MGR (*p*-value = 0.684; Supplementary Table S5a-c). These results are consistent when using the subset of the data that only contained the pairings with significantly higher colonization in the inoculated seedlings (Supplementary Table S5j-l). The presence of hyphae and conidiophores was also unrelated to MGR (Supplementary Table S5d-i).

### Mycorrhizal growth response

**Fig. 3:**
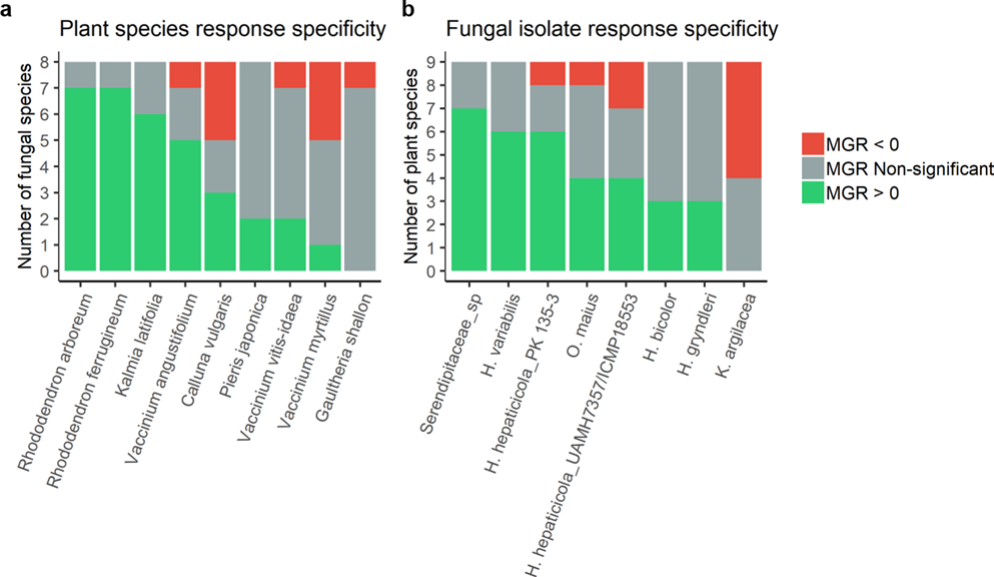
Response specificity of ericoid mycorrhizal fungal isolates and their host plants. Each bar shows how many symbiotic partners with which each plant species (**a**) or fungal isolate (**b**) produced or exhibited a positive mycorrhizal growth response (MGR), negative MGR, or non-significant MGR for total biomass of the plants studied. Species exhibiting/producing a positive MGR with only a small number of symbiotic partners are considered to have high response specificity, whereas those producing/exhibiting a positive MGR with many partners are considered response generalists

MGR was positive or neutral for most plant species, but also occasionally negative (Fig. 2b, total biomass). Three plant species (*K. latifolia*, *R. arboreum*, and *R. ferrugineum*) showed “generalist” tendencies, or low growth response specificity, with growth responding positively to almost all fungal isolates (Figs. 2b and 3a). Inoculated seedlings of these plant species had 2.46, 1.99, and 2.97 times more biomass than control seedlings across all fungal treatments, respectively (*p*-value < 0.0001; Supplementary Table S2d). In contrast, *V. myrtillus* showed relatively higher response specificity, only significantly benefiting from *Serendipitaceae* sp. JPK 132 (Fig. 2b and Supplementary Table S2b). Additionally, *V. myrtillus* was significantly negatively affected by *H. hepaticicola* PK 135-3, *H. hepaticicola* UAMH 7357, and *Ku. argillacea* JPK 87 (Fig. 2b and Supplementary Table s2Bb). The remaining plant species showed moderate levels of response specificity, with total biomass responding positively to some fungal isolates but not others (Figs. 2b and 3a). Above and belowground MGR showed similar patterns to total biomass (Supplementary Figure S3d and S4d).

From the fungal perspective, *Serendipitaceae* sp. JPK 132, *H. variabilis* UAMH 11,265, and *H. hepaticicola* PK 135-3 showed low growth response specificity, significantly increasing the growth of at least six of the plant species (Figs. 2b and 3b). These ErMF also consistently showed the highest levels of MGR across all plants, with the mean MGR across plant species ranging between 1.32 and 2.45 (*p*-value < 0.0001; Supplementary Table S2e). Findings were consistent for above and belowground MGR (Supplementary Table S3e and S4e). Conversely, *Ku. argillacea* JPK 87 did not show any beneficial effect, except for increasing the belowground biomass of *K. latifolia* (Supplementary Figure S4b). In five cases, *Ku. argillacea* JPK 87 even negatively influenced total biomass (Fig. 2b, Supplementary Table S2b). The most beneficial plant-fungal combination was the inoculation of *R. ferrugineum* with *H. bicolor* UAMH 11,274, leading to a total biomass increase of 12 times if compared to the control (Fig. 2b, Supplementary Table S2b).

There did not appear to be strong similarities in MGR at the fungal genus level. Although not directly tested, the five isolates from the *Hyaloscypha* genus did not seem to produce more similar MGRs than isolates from other genera. However, the two fungal isolates from the same species, *H. hepaticicola* PK 135-3 and *H. hepaticicola* UAMH 7357, did show similar patterns of MGR, except when paired with *C. vulgaris*. In this case, *H. hepaticicola* PK 135-3 produced a positive MGR, but *H. hepaticicola* UAMH 7357 was detrimental (Fig. 2b, Supplementary Fig. S4a and Supplementary Tables S2b-c and S4b-c).

### Shoot:root biomass allocation

**Fig. 4:**
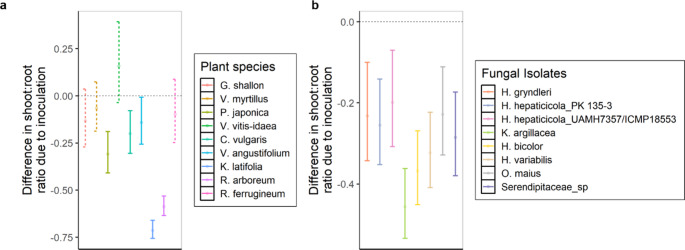
Plants allocate more resources to root development in the presence of ericoid mycorrhizal fungi. Plots show the difference in shoot:root biomass ratio due to mycorrhizal inoculation estimated marginal mean values (circle) and the 95% confidence interval for each plant species across all fungal treatments (**a**) and fungal treatment across all plant species (**b**). Values below zero indicate that plants were allocating more resources to the roots when inoculated with ericoid mycorrhizal fungi compared to the control, and values above zero indicate more resources were allocated to the shoots when inoculated compared to the control. Non-significant shoot:root biomass allocation ratios are represented by dashed lines. *n* = 631, ranging from 4 to 10 replicates per plant-fungal unique combination, except for *K. latifolia*–*H. gryndleri* MGR-3 and *R. ferrugineum*–*Ku. argillacea* JPK 87 which only have 2 replicates

Resource allocation was dependent on both the fungal identity (*p*-value < 0.01) and plant species (*p*-value < 0.001), but not on their interaction (*p*-value = 0.218, Supplementary Table S6b). All fungal treatments resulted in a significant shift in allocation of plant resources towards the roots when compared to the control seedlings (Fig. 4b). For plant species, the same trend was observed, as inoculation resulted in the preferential allocation of resources towards the roots compared to control plants. However, shoot: root ratio allocation was not significantly different to the control for four species (*G. shallon*, *V. myrtillus*, *V. vitis-idaea*, and *R. ferrugineum*) (Fig. 4a).

### Plant phylogenetic signal

**Fig. 5:**
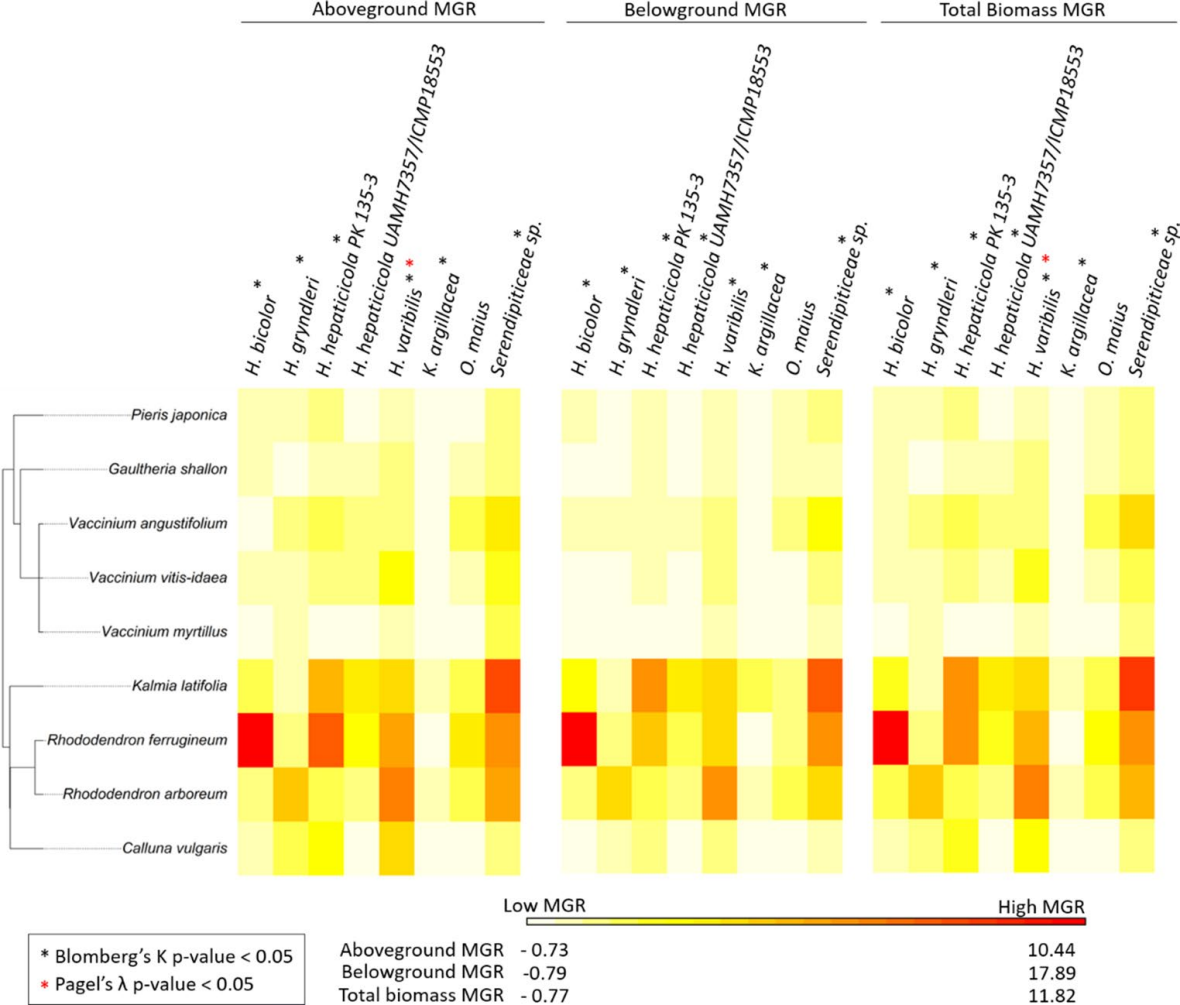
Evidence of plant phylogenetic signal in mycorrhizal growth response. The heat map represents the hierarchical clustering of the mycorrhizal growth response (MGR) of each plant species when inoculated with ericoid mycorrhizal fungi for above, belowground, and total biomass. The asterisks (black for Blomberg’s K and red for Pagel’s λ) indicate significant phylogenetic signal (Blomberg’s K > 1.00, Pagel’s λ close to 1.00, *p*-value: < 0.05 *). *n* = 631, ranging from 4 to 10 replicates per plant-fungal unique combination, except for *K. latifolia*–*H. gryndleri* MGR-3 and *R. ferrugineum*–*Ku. argillacea* JPK 87 which only have 2 replicates

We found evidence that plant phylogeny impacted MGR across most fungal species (Fig. 5). Specifically, Blomberg’s K was significant for aboveground, belowground, and total biomass MGRs for all fungal isolates (*p*-value < 0.038), except for *O. maius* JPK 11 (p-value > 0.052) and for aboveground biomass only for *H. hepaticicola* UAMH 7357 (*p*-value = 0.099). Pagel’s λ was also significant for *H. variabilis* UAMH 11,265 for aboveground (*p*-value < 0.05), and total biomass MGRs (*p*-value < 0.05; Supplementary Table S7).

## Discussion

In this study, we expanded baseline knowledge of the effects of the ErM symbiosis on plant growth by screening the MGR of 72 unique combinations of nine ericaceous plant species and eight ErMF isolates. Our results showed that fungal and plant identity influenced MGR, with different species/isolates showing different levels of response specificity. Additionally, we report the existence of a phylogenetic signal for MGR in the presence of almost all fungal species tested. Finally, the finding that colonization levels were unrelated to MGR supports results of previous studies (Guo et al. 2022; Lutz et al. 2023; Ma et al. 2024; Thirkell et al. 2022; Treseder 2013), and highlights that colonization intensity is not indicative of mycorrhizal growth benefit. Interestingly, inoculation with all isolates led to a preferential allocation of resources towards the roots compared to control plants. Plant biomass allocation to leaves, roots, and shoot has important implications for the carbon residence time in forests, and greater biomass allocation to the root system may increase soil carbon stocks in ErM habitats (Jevon and Lang 2022). These findings comprise valuable information for a better understanding of the ErM symbiosis as well as for restoration and conservation efforts.

### The generalist-specialist continuum

Our experiment revealed that ericaceous plants can be either specialists or generalists regarding the mycorrhizal benefit they gain from ErMF. The only response specialist plant detected was *V. myrtillus*, which responded positively to only one fungal isolate (*Serendipitaceae* sp. JPK 132). Conversely, *K. latifolia*, *R. arboreum*, and *R. ferrugineum* were response generalists, responding to either six or seven fungal isolates. The particularly high response specificity of *V. myrtillus* shown here was surprising, as previous studies found that *V. myrtillus* produced greater biomass upon inoculation with *O. maius* (Casarrubia et al. 2020; Martino et al. 2018). That *V. myrtillus* did not respond positively to *O. maius* in our study might be due to differences in the experimental design, such as the fungal-plant co-cultivation system set-up and time span, the substrate used, or the utilization of isolates with specific genotypes that may differ in their association with the host. Therefore, more research is needed to determine what environmental factors determine such response specialization.

We found that ErMF also fall within the generalist-specialist continuum when assessing the growth benefit they provide to ericaceous plants. All fungal isolates resulted in a beneficial response for at least three plant species, with no species exhibiting high specificity. *Kurtia argillacea* was the only exception, consistently having neutral or negative effects on the investigated plant species. Mycorrhizal fungi can show parasitic tendencies if net costs to the plants of maintaining the association exceed net benefits. This can occur at certain developmental stages, particularly when seedlings are young, or under certain environmental conditions or between pairings of certain genotypes (Johnson et al. 1997). For example, a large-scale field inoculation trial of maize plants with AM fungi resulted in a highly variable growth response to AM fungi inoculation, ranging from − 12% to + 40% (Lutz et al. 2023). These results highlight the complexity of mycorrhizal interactions and the importance of substrate type, nutrient availability, microclimatic conditions, plant and/or fungal co-cultivation time span, as well as the plant-fungal pairing in determining plant growth outcome (Martin and van der Heijden 2024).

Five of the fungal isolates in this study were able to provide benefit to four or more plant species, showing low response specificity. This could indicate that many ErMF species are generalists, but could also be the result of isolation biases influencing the pool of ErMF tested (Johnson et al. 2005). Some ErMF are difficult to isolate, and it is possible that generalist species may be more easily isolated, consequently dominating the currently available culture collections. Future efforts to expand available ErMF cultures should improve the range of ErMF species available to be included in greenhouse studies. Additionally, culture-independent methods, such as metabarcoding analyses of ErMF in natural and semi-natural environments, should also be used to better understand ErM specificity and growth benefits (Chen et al. 2021; Vohník et al. 2023; Yurgel et al. 2018).

Increasing the range of plant and fungal species screened in this experiment allowed a modest, but meaningful, expansion in the field of ErM symbiosis research. However, future work should test MGR of more plants to a larger range of fungal species to better represent the full spectrum of ErM plant and fungal species that exist (Van Geel et al. 2020; Vohník et al. 2023). It will be especially important to compare co-occuring (i.e. coevolved) vs. naïve plant-fungal combinations. In our study, all of the fungal isolates originated from Europe, whereas plant species originated from across the globe. Although we do not have enough representation to statistically test differences in MGR between co-evolved and naïve plant-fungal combinations, we suggest that co-evolved combinations may show stronger MGR. Specifically targeting naïve combinations will help inform potential shifts in mycorrhizal benefit resulting from invasions. Further work should also assess plant response specificity using metrics other than MGR, such as those that can measure water uptake ability, drought resistance, or ability to cope with heavy metal toxicity. Indeed, in ErM habitats, due to the acidic soil conditions, toxic metal ions can be highly available (Meharg and Cairney 1999), with evidence that ErMF provide host plant protection (Bradley et al. 1981; Sharples et al. 2000). Finally, it would be of interest to focus on the fungus benefit rather than solely on plant benefit to better understand specificity in the ERM symbiosis.

### Plant phylogenetic signal

That MGR of seven out of eight fungal isolates showed plant phylogenetic signal in this study indicates that plant evolutionary history influences plant response specificity to ErMF. Numerous studies in AM and EcM systems have found that plant phylogeny is a strong predictor of plant–fungal association specificity and the overall community composition of fungal species that colonize host roots (Botnen et al. 2020; Põlme et al. 2013; Reinhart et al. 2012; Tedersoo et al. 2013; van Galen et al. 2023). A meta-analysis across AM and EcM plant response to mycorrhizal fungi also found that plant and fungal evolutionary history explained considerably more variation in the response than numerous ecological factors (Hoeksema et al. 2018). Our results indicate that plant evolutionary history is also important for MGR in ErM systems.

### Implications for conservation and restoration

Together, our results suggest important implications for conservation and restoration efforts. Response specificity is particularly important, as plant species with high response specificity might be more restricted to the exact fungal species they benefit from or be more sensitive to environmental change that impacts their fungal symbionts. Conversely, response generalists may be more resistant. Therefore, in sites with a high abundance of generalist plants, it might be easier to manage restoration approaches due to a less restrictive set of fungal inoculum options that can be used. While in this project, we used plant species with broad native ranges, in small-scale management projects, locally relevant generalist species should be considered to avoid unanticipated negative consequences. In particular, care must be taken to mitigate any potential negative outcomes of inoculation, such as the inadvertent introduction of non-native fungal species (Dickie et al. 2017; Thomsen and Hart 2018). Moreover, is has been shown that native fungal communities result in high success rates for native plants (Koziol et al. 2018), as native fungal taxa have coevolved with resident plant species and/or populations (Brundrett 2002; Delavaux and Bever 2022; Hoeksema et al. 2018).

Additionally, the evidence that plant genetic relatedness influences MGR indicates that the plant phylogeny shows promise in predicting mycorrhizal growth response of plants not included in this study (Reinhart et al. 2012). This could assist restoration and conservation efforts by avoiding the need to experimentally determine MGR of all species in an ecosystem. However, the ErM core Ericaceae comprises five subfamilies (Vohník 2020), so a much wider variety of plants should be examined before the predictive accuracy the host phylogeny can be properly evaluated and implemented.

## Conclusions

Our study sheds new light on the ErM symbiosis by providing information on its formation and benefit across a wider range of both plant and fungal species than previously investigated. This gives insights regarding the importance of both plant and fungal partner identity on the formation and function of ErM associations, with potentially important implications for management and restoration strategies. Finally, we also found evidence of a plant phylogenetic signal, which could potentially help to predict the growth response of ericaceous species not yet included in greenhouse experiments. Ultimately, this work advances our fundamental understanding of plant growth response benefit to ErMF and specificity of this benefit across a greater number of plant hosts. This type of effort will be vital to improving our ability to manage and restore wetland and heathland systems that depend on this understudied symbiosis.

## Supplementary Information

Below is the link to the electronic supplementary material.

Supplementary file1 (DOCX 2.10MB)

Supplementary file2 (XLSX 184KB)

Supplementary file3 (XLSX 316KB)

Supplementary file4 (PDF 632KB)

## Data Availability

All data and code is available at GitHub (https://github.com/asimoesneves/Ericoid-mycorrhizal-growth-response-is-influenced-by-host-plant-phylogeny).
