# Supplementary material for: Ericoid mycorrhizal growth response is influenced by host plant phylogeny
Source: Mycorrhiza. 2025 Aug 13;35(4):51. doi: 10.1007/s00572-025-01223-6 (PMC12350526; doi:10.1007/s00572-025-01223-6)
Supplement: Supplementary file 1 — Supplementary file1 (DOCX 2.10MB) [file 572_2025_1223_MOESM1_ESM.docx]

# Supplementary Materials

## **Supplementary Text 1. DNA extraction and Sanger sequencing**

Mycelium was sampled from agar plates, lyophilized and ground before DNA extraction using the sbeadex technology with a customized extraction protocol for plant material (LGC Genomics, Berlin, Germany) on an automated KingFisher 96/Flex instrument (ThermoFisher Scientific, Basel, Switzerland). DNA was dissolved in 100 µl AMB buffer (LGC Genomics, Berlin, Germany) and stored at -20°C until Sanger Sequencing.

We amplified the ITS region of the nuclear ribosomal internal transcribed spacer (ITS) region using the reverse primer ITS4 and one of the forward primers ITS1F, ITS1, or ITS5 for the strains *H. hepaticicola* PK 135-3, *Oidiodendron maius* JPK 11, and *Kurtia* (*Ku.*) *argillacea* JPK 87, respectively (Gardes & Bruns, 1993; White et al., 1990). We applied the following conditions for PCRs: for a total volume of 20 µl, we used 3 µl of a 1:10 dilution of DNA, the GoTaq G2 Green Mastermix (Promega, Dübendorf, Switzerland) with cycling conditions of 2 min at 94°C of initial denaturation, 34 cycles of 30 sec at 94°C denaturation, 30 sec at 56°C annealing, and 2 min at 72°C elongation, and a final elongation of 10 min at 72°C. PCR products were enzymatically purified using Exonuclease I and FastAP Thermosensitive Alkaline Phosphatase according to the manufacturer's instructions (ThermoFisher Scientific, Basel). Cycle sequencing was performed using the BigDye Terminator v3.1 Cycle Sequencing Kit and products were purified using the BigDye XTerminator purification kit (ThermoFisher Scientific, Basel) before analysis on an Applied Biosystems ABI-3500 genetic analyzer. The raw sequencing data was visualized and processed using CLC MainWorkbench 7.9.1 (Qiagen, Stockach).

**References**

Gardes M, Bruns TD (1993) ITS primers with enhanced specificity for basidiomycetes—Application to the identification of mycorrhizae and rusts. Mol Ecol 2(2):113–118. <https://doi.org/10.1111/j.1365-294X.1993.tb00005.x>

White TJ, Bruns T, Lee S, Taylor J (1990) 38-Amplification and direct sequencing of fungal ribosomal RNA genes for Phylogenetics. In PCR Protocols (pp. 315–322). Elsevier. <https://doi.org/10.1016/B978-0-12-372180-8.50042-1>


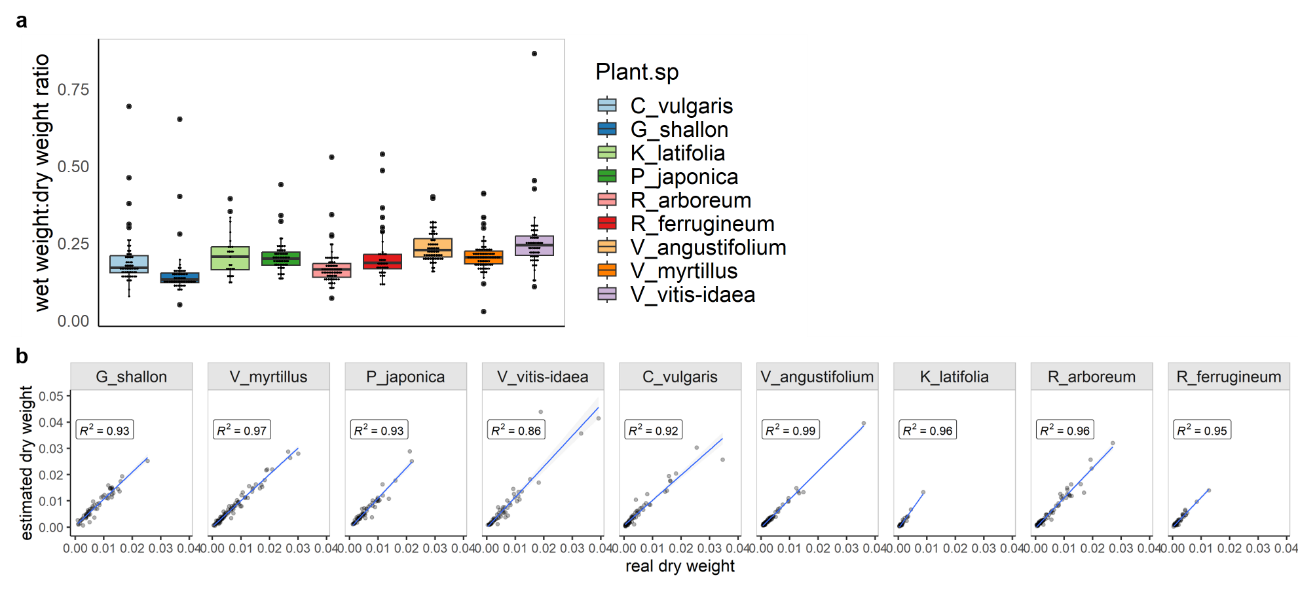


**Figure S1. Wet weight:dry weight ratio of roots from nine ericaceous plant species used in this study.** Wet weight:dry weight ratio per plant species (a) and comparison between the real dry weight of the samples used to calculate the wet weight:dry weight ratio and the estimated values for dry weight of the same samples using the ratio (b). One outlier was removed for *Vaccinium myrtillus*. n = 62, 60, 27, 61, 73, 41, 71, 87, and 60 (*C. vulgaris*, *G. shallon*, *K. latifolia*, *P. japonica*, *R. arboreum*, *R. ferrugineum*, *V. angustifolium*, *V. myrtillus*, and *V. vitis-idaea*, respectively).


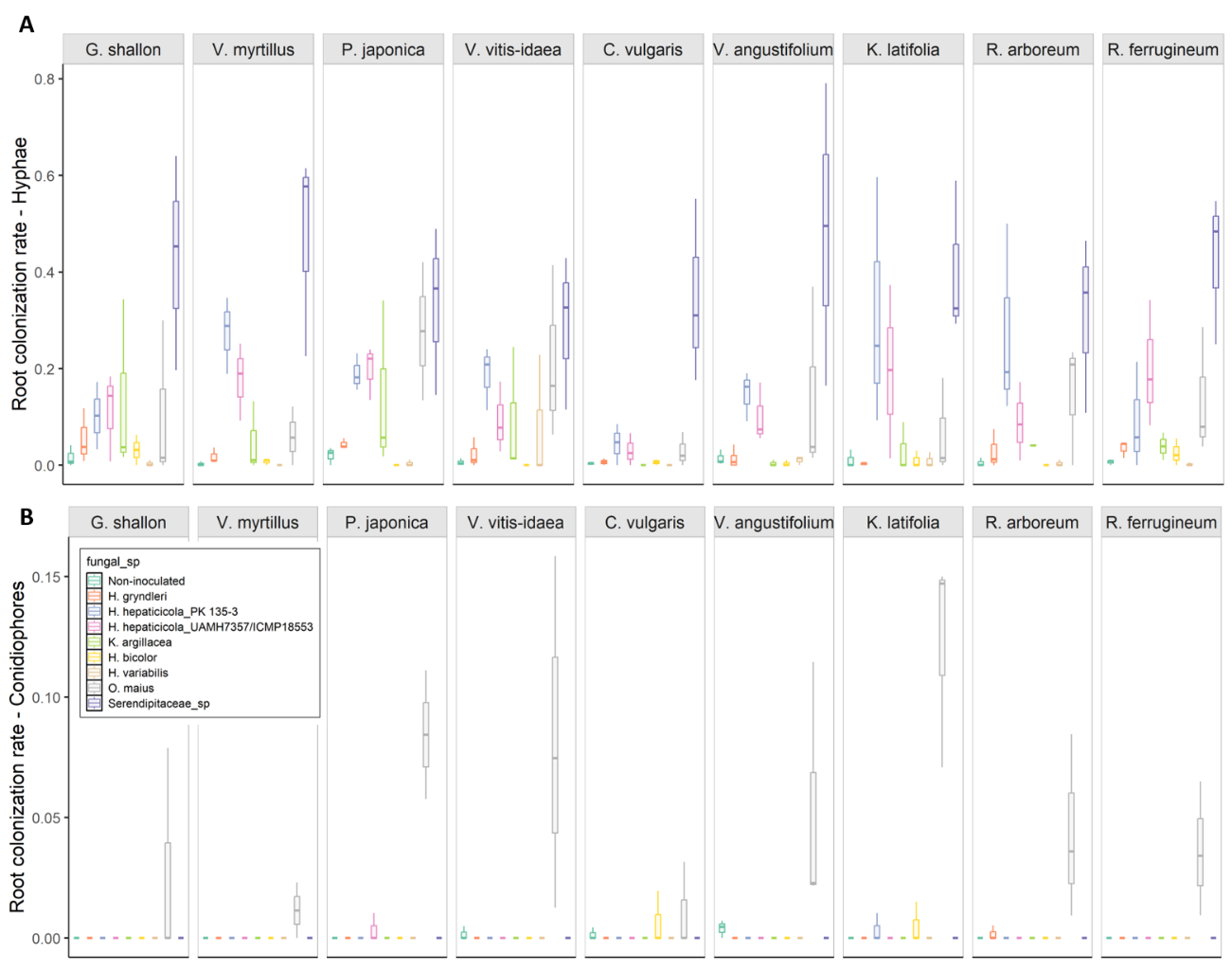


**Figure S2. Proportion of plant root cells that present ErM structures.** Proportion of hyphae (a) and conidiophores (b) present in each plant-fungi combination. n = 239, three replicates per unique combination were subsampled and stained to be scored for the presence of fungal coils, except for *K. latifolia*–*H. gryndleri* MGR-3, *C. vulgaris*–*H. gryndleri* MGR-3, *P. japonica*–*O. maius* JPK 11, *G. shallon*–*H. hepaticicola* PK 135-3, and *R. ferrugineum*–*Ku. argillacea* JPK 87, which only have two replicates and *V. angustifolium*–*H. gryndleri* MGR-3, which has four replicates.


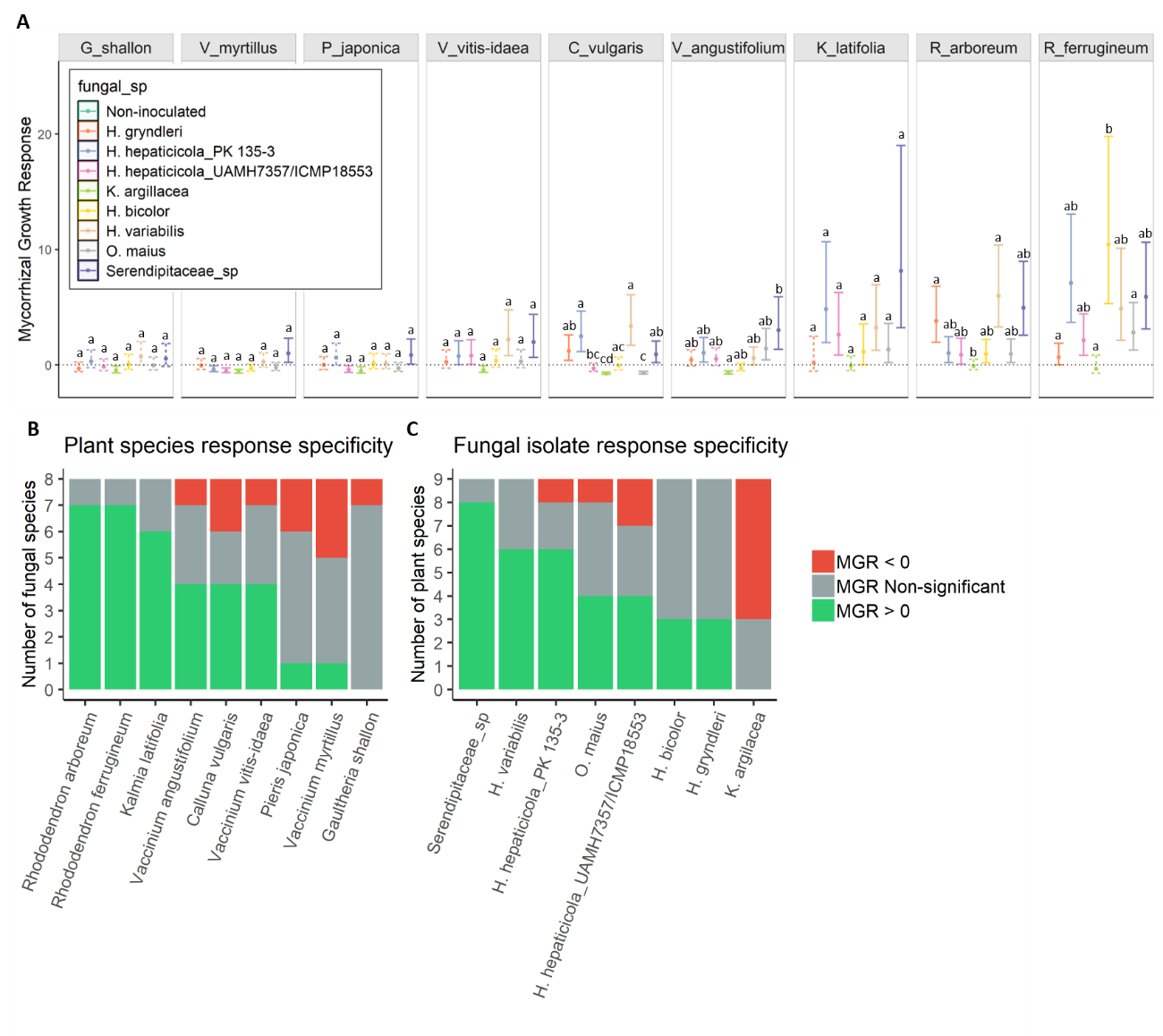


**Figure S3. Aboveground mycorrhizal growth response and plant and fungal response specificity.** Mycorrhizal growth response (MGR) for aboveground biomass of the plants studied (a). Plots show MGR estimated marginal mean values (circles) and the 95 % confidence intervals for each plant-fungi unique combination. Non-significant MGRs (where confidence intervals include zero) are represented by dashed lines. Within the same plant species, significantly different MGRs have different letters. n = 631, ranging from 4 to 10 replicates per plant-fungal unique combination, except for *K. latifolia*–*H. gryndleri* MGR-3 and *R. ferrugineum*–*Ku. argillacea* JPK 87, which only have 2 replicates. Response specificity of plants (b) and fungi (c) for aboveground biomass. Number of species that show a positive, negative and non-significant MGR are green, red and grey respectively (Supplementary Table S8a). Species producing/exhibiting a positive MGR with only a small number of symbiotic partners are considered to have high response specificity, whereas those producing/exhibiting a positive MGR with many partners are considered response generalists.


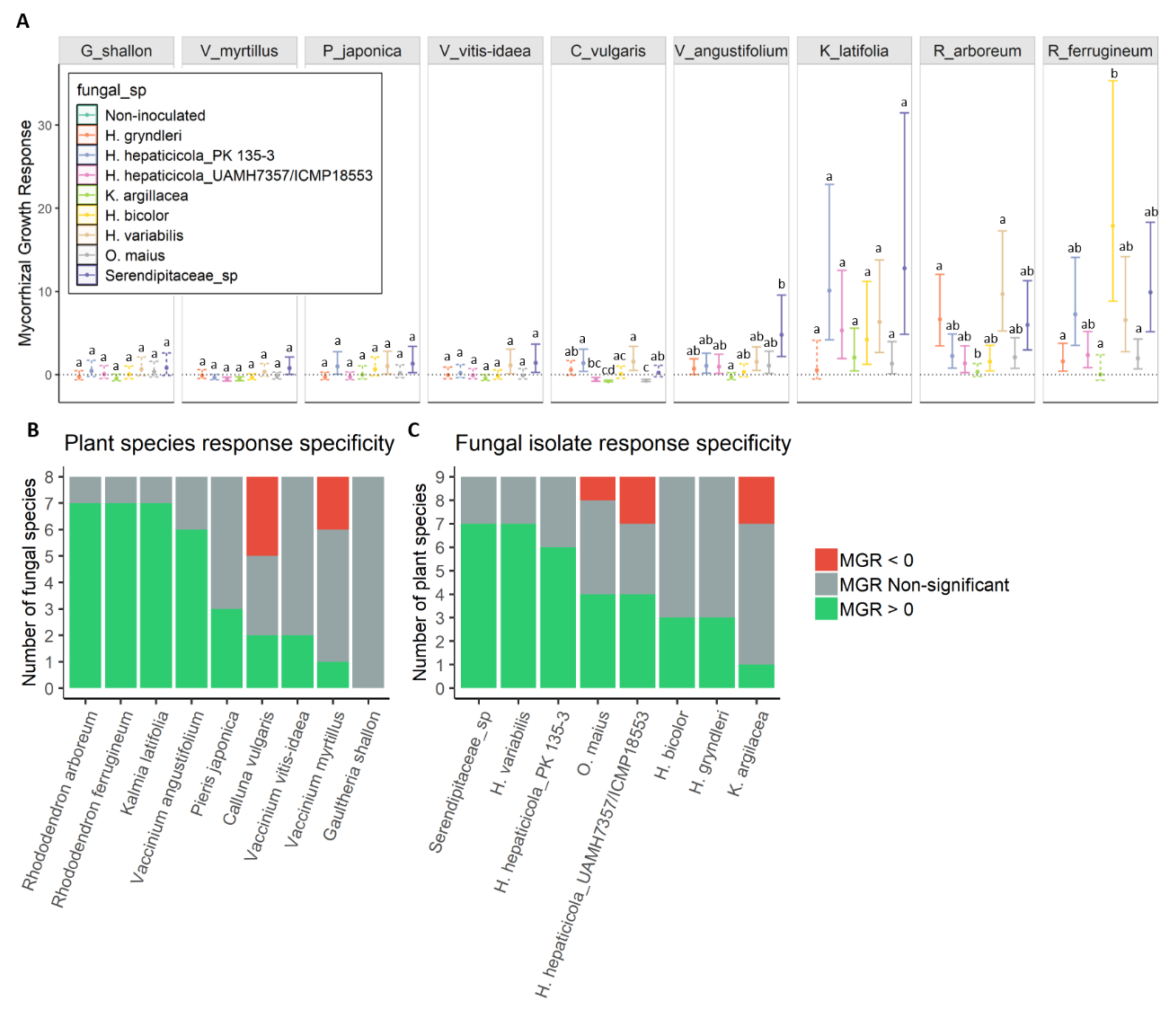


**Figure S4. Belowground mycorrhizal growth response and plant and fungal response specificity.** Mycorrhizal growth response (MGR) for belowground biomass of the plants studied (a). Plots show MGR estimated marginal mean values (circles) and the 95 % confidence intervals for each plant-fungi unique combination. Non-significant MGRs (where confidence intervals include zero) are represented by dashed lines. Within the same plant species, significantly different MGRs have different letters. n = 631, ranging from 4 to 10 replicates per plant-fungal unique combination, except for *K. latifolia*–*H. gryndleri* MGR-3 and *R. ferrugineum*–*K. argillacea* JPK 87, which only have 2 replicates. Response specificity of plants (b) and fungi (c) for belowground biomass. Number of species that show a positive, negative and non-significant MGR are green, red and grey respectively (Supplementary Table S8b). Species producing/exhibiting a positive MGR with only a small number of symbiotic partners are considered to have high response specificity, whereas those producing/exhibiting a positive MGR with many partners are considered response generalists.
