# Supplementary material for: Ericoid mycorrhizal growth response is influenced by host plant phylogeny
Source: Mycorrhiza. 2025 Aug 13;35(4):51. doi: 10.1007/s00572-025-01223-6 (PMC12350526; doi:10.1007/s00572-025-01223-6)
Supplement: Supplementary file 4 — Supplementary file4 (PDF 632KB) [file 572_2025_1223_MOESM4_ESM.pdf]

Title:Ericoid mycorrhizal growth response is influenced by host plant phylogeny

Authors: Alice S. Neves, Laura G. van Galen, Martin Vohník, Martina Peter, Elena Martino, Thomas W. Crowther, Camille S. Delavaux

================================================================================

1. Data processing.R

- In this R script the dry root biomass is calculated using a conversion ratio obtained with the dry root biomass data available from the samples that we were able to weight using the scale (wet weight : dry weight ratio).

- The total dry biomass is calculated as well as the mycorrhizal growth response (MGR) for above, belowground and total biomass. The shoot : root dry weight ratio and the difference between shoot : root dry weight ratio between treated and control seedlings (here as MGR ratio) was also calcuated. These values are added to the master dataframe that is exported as a csv table and will be further used in the next R scripts.

--------------------------------------------------------------------------------

2. differences_estimates.R

- the difference between the real dry biomass values and the estimates originated using the wet weight : dry weight ratio are also plotted with the R2 values to check if the ratio is aproppriate to be used

--------------------------------------------------------------------------------

3. colonization_assessment.R

- In this R script the proportion and percentage of colonized roots is calculated for fungal coils, hyphae and conidiophores (spikes)

- Proportion of colonization is added to the master dataframe

- The efficiency of the inoculation is tested by analysing if the colonization of treated samples is significantly higher than the controls

- we were only able to do so for hyphal coils

- hyphae and conidiophores have raw data plotted

- It was tested if mycorrhizal growth response (MGR) can be predicted by proportion of colonization

--------------------------------------------------------------------------------

4. plant_models_MGR.R

- In this R script the MGR is modelled using linear mixed effects models

- marginal square means and pairwise comparisons between plant-fungi combinations

- model for shoot : root ratio MGR

--------------------------------------------------------------------------------

5. response_specificity_plots.R

- generate plots of response specificy for fungi and plant regarding above, belowground and total MGR

--------------------------------------------------------------------------------

6. ericoid_phylogeny.R

- In this R script a phylogenetic tree for the plant species used in the experiment is obtained from the GBOTB

- The MGR is ploted in a heath map and the existence of phylogenetic signal for the MGR in the plant phylogeny is tested (using Blomberg's K and Pagel's lambda)

# 1. Data processing.R

```
library(tidyverse)

library(ggplot2)

library(ggsci)

library(RColorBrewer)

library(lme4)

library(lmtest)

library(lmerTest)

library(sjPlot)


###### In this R script, the dry root biomass is calculated using the conversion ratios;

########### the total biomass and MGR are also calculated and everything is added to the

########### dataframe that is exported as a csv table ("Harvested_Plants_Processed.csv")

########### and is ready to be used for further modelling


dat_og = read.csv2("measurements_21stFebruary_Version.csv")

str(dat_og)


dat_og = dat_og[1:714, 1:9]


dat_og =dat_og %>%select(-c("Height.t1"))


dat2 = read.csv2("Harvested_Plants_june.csv")



str(dat2)


dat2 = dat2[1:643, 1:19]


dat2 = dat2 %>% select(-c("plant_sp", "fungal_sp", "replicate", "treatment",
                  "block", "Height.t0", "t1.t0", "Dead.Sick", "X.cassette"))


str(dat2)


dat = dat_og %>%
```

```
  left_join(dat2, by = "unique_id")

str(dat)

##### CHANGE PLANT SP NAMES FOR LONG NAME

dat <- transform(dat,plant_sp=gsub(pattern="CVU", replacement="C. vulgaris", plant_sp))
dat <- transform(dat,plant_sp=gsub(pattern="GSH", replacement="G. shallon", plant_sp))
dat <- transform(dat,plant_sp=gsub(pattern="KLA", replacement="K. latifolia", plant_sp))
dat <- transform(dat,plant_sp=gsub(pattern="PJA", replacement="P. japonica", plant_sp))
dat <- transform(dat,plant_sp=gsub(pattern="RAR", replacement="R. arboreum", plant_sp))
dat <- transform(dat,plant_sp=gsub(pattern="RFE", replacement="R. ferrugineum", plant_sp))
dat <- transform(dat,plant_sp=gsub(pattern="VAN", replacement="V. angustifolium", plant_sp))
dat <- transform(dat,plant_sp=gsub(pattern="VMY", replacement="V. myrtillus", plant_sp))
dat <- transform(dat,plant_sp=gsub(pattern="VVI", replacement="V. vitis-idaea", plant_sp))


##### CHANGE FUNGAL SP NAMES FOR LONG NAME

dat <- transform(dat,fungal_sp=gsub(pattern="JPK-132", replacement="Serendipitaceae_sp", fungal_sp))
dat <- transform(dat,fungal_sp=gsub(pattern="JPK-87", replacement="K. argillacea", fungal_sp))
dat <- transform(dat,fungal_sp=gsub(pattern="MEB", replacement="H. bicolor", fungal_sp))
dat <- transform(dat,fungal_sp=gsub(pattern="MEV", replacement="H. variabilis", fungal_sp))
dat <- transform(dat,fungal_sp=gsub(pattern="MGK", replacement="H. gryndleri", fungal_sp))
dat <- transform(dat,fungal_sp=gsub(pattern="OMA", replacement="O. maius", fungal_sp))
dat <- transform(dat,fungal_sp=gsub(pattern="RER", replacement="H. hepaticicola_PK 135-3", fungal_sp))
dat <- transform(dat,fungal_sp=gsub(pattern="RHE", replacement="H. hepaticicola_UAMH7357/ICMP18553", fungal_sp))
dat <- transform(dat,fungal_sp=gsub(pattern="STR", replacement="Sterile", fungal_sp))

############################################################################################
########################################  RATIO  ##########################################
############################################################################################

##calculate average wet/dry biomass ratio for each plant_sp
```

```
dat1 <- data.frame()

# add real values of wet and dry weight from experiment to the table

#need to do in 2 steps: first until row 243 - where largesubsample was done

ratio_real_values <- filter(dat, random_label<=243)

ratio_real_values = ratio_real_values %>% select(-c("unique_id", "fungal_sp", "replicate", "treatment",
          "block", "Height.t0", "Dead.Sick", "random_label", "Height.t1",
          "blockx", "total_root_wet", "small_subsample_wet", "total_root_dry",
          "shoot_weight"))

colnames(ratio_real_values)[colnames(ratio_real_values) == c("plant_sp", "large_subsample_wet","large_subsample_dry") ] <- c("Plant.sp", "weight.wet.root", "weight.dry.root")

dat1 <- rbind(dat1, ratio_real_values)

# and then from 244 until the end with total_root_wet and total_root_dry

ratio_real_values1 <- filter(dat, random_label>243)

ratio_real_values1 = ratio_real_values1 %>% select(-c("unique_id", "fungal_sp", "replicate", "treatment",
                    "block", "Height.t0", "Dead.Sick", "random_label", "Height.t1",
                    "blockx", "large_subsample_wet", "small_subsample_wet", "large_subsample_dry",
                    "shoot_weight"))

colnames(ratio_real_values1)[colnames(ratio_real_values1) == c("plant_sp", "total_root_wet","total_root_dry") ] <- c("Plant.sp", "weight.wet.root", "weight.dry.root")

dat1 <- rbind(dat1, ratio_real_values1)

#group by

#mean (in tidyverse)

dat1 = dat1 %>% drop_na()

dat1$ratio = dat1$weight.dry.root/dat1$weight.wet.root

xx=dat1%>%
 group_by(Plant.sp)%>%
```

```
  summarize(Mean = mean(ratio))

ratio_table = data.frame(xx)
colnames(ratio_table) <- c("plant_sp", "ratio")

#ratio_table$percentage_ratio = ratio_table$ratio*100
write.csv2(ratio_table, "ratio_table.csv", row.names = FALSE)

png("figures/biomass_ratio/Distribution_ratio_dots_updated1.jpg", width = 8, height = 3.5, units ='in', res = 300)
ggplot(dat1, aes(x=Plant.sp, y=ratio)) +
  geom_boxplot(aes(fill=Plant.sp)) +
  scale_fill_brewer(palette = "Paired") +
  geom_dotplot(binaxis='y', stackdir='center', dotsize=0.5, binwidth = 0.011) +
  ggtitle(label = "Distribution of ratio dry/wet per plant sp") +
  theme_minimal(base_size = 15) +
  theme(
    panel.background = element_rect(fill = NA, color = "grey", size = 0.5),
    panel.grid.major = element_blank(),
    panel.grid.minor = element_blank(),
    plot.background = element_rect(fill = "transparent", colour = NA_character_),
    strip.background = element_rect(fill = "grey90", color = "grey", size = 0.5),
    axis.title.x = element_blank(),
    axis.text.x = element_blank(),
    axis.ticks.x = element_blank(),
    axis.line = element_line(size = 0.5),
    strip.text = element_text(size = 15),
    plot.title = element_text(hjust = 0.5, size = 16),
    axis.title.y = element_text(size = 16),
    legend.title = element_text(size = 17),
    legend.text = element_text(size = 16)
  ) +
  labs(y= "wet weight:dry weight ratio") +
  theme(axis.title.x=element_blank())
dev.off()
```

```
##########################################################################################
########################### root biomass calculations ###################################
##########################################################################################

#calculate small subsample wet weight
dat$small_subsample_wet = dat$total_root_wet-dat$large_subsample_wet

# calculate dry biomass for large subsample - those that the scale didn't detect
### use average ratio!


#join ratio in dataframe of main excel spreadsheet and
ratio_avrg = read.csv2("ratio_table.csv")
dat = dat %>% left_join(ratio_avrg)
#dat = dat %>% select(-c("percentage_ratio"))

i1 <- is.na(dat$large_subsample_dry)
dat$large_subsample_dry[i1] <- with(dat, large_subsample_wet[i1] *ratio[i1])


# calculate dry biomass for small subsample
## 1. if don't have large subsample -> use average ratio
## 2. if have large subsample -> use equation
dat = dat %>% drop_na(large_subsample_wet)
for (i in 1:630){
  if (dat$large_subsample_wet[i] == 0) {
    dat$small_subsample_dry[i] = dat$small_subsample_wet[i]*dat$ratio[i]
  }
  else {dat$small_subsample_dry[i] =
    (dat$large_subsample_dry[i]/dat$large_subsample_wet[i])*dat$small_subsample_wet[i]}
}


i2 <- is.na(dat$total_root_dry)
```

```
dat$total_root_dry[i2] <- with(dat, dat$small_subsample_dry[i2]+dat$large_subsample_dry[i2])



################################################################################
###############################  BIOMASS  #####################################
################################################################################
######################TOTAL BIOMASS, MGR CALCULATION###########################
################################################################################
################################################################################

##MGR FOR ABOVEGROUND
STR=dat[dat[,"fungal_sp"]=="Sterile",c("plant_sp","shoot_weight")]

avr_STR = rep(0, times=9)
plant_nam = c("C. vulgaris", "G. shallon", "K. latifolia", "P. japonica", "R. arboreum",
      "R. ferrugineum", "V. angustifolium", "V. myrtillus", "V. vitis-idaea")

for (i in 1:9){
 x = STR[STR[,"plant_sp"]==plant_nam[i],]
 avr_STR[i] = mean(x[,"shoot_weight"], na.rm=TRUE)
}

abvg_biomass_STR_table = data.frame(plant_sp=plant_nam, abvg_biomass_STR=avr_STR)

###left join in dat
dat = dat%>%left_join(abvg_biomass_STR_table)

###calculate MGR = (Biomass EMF - Biomass STR)/Biomass STR in new column in the dataframe

dat$MGR_abvg = (dat$shoot_weight-dat$abvg_biomass_STR)/dat$abvg_biomass_STR



##MGR FOR BELOWGROUND
STR_b=dat[dat[,"fungal_sp"]=="Sterile",c("plant_sp","total_root_dry")]
```

```
avr_STR_b = rep(0, times=9)
plant_nam = c("C. vulgaris", "G. shallon", "K. latifolia", "P. japonica", "R. arboreum",
        "R. ferrugineum", "V. angustifolium", "V. myrtillus", "V. vitis-idaea")

for (i in 1:9){
  x = STR_b[STR_b[,"plant_sp"]==plant_nam[i],]
  avr_STR_b[i] = mean(x[,"total_root_dry"], na.rm=TRUE)
}

blwg_biomass_STR_table = data.frame(plant_sp=plant_nam, blwg_biomass_STR=avr_STR_b)

###left join in dat
dat = dat%>%left_join(blwg_biomass_STR_table)

###calculate MGR = (Biomass EMF - Biomass STR)/Biomass STR in new column in the dataframe

dat$MGR_blwg = (dat$total_root_dry-dat$blwg_biomass_STR)/dat$blwg_biomass_STR



#calculate total biomass = root + shoot (below and aboveground)

dat$total_biomass = dat$total_root_dry + dat$shoot_weight

#calculate average total biomass for sterile treatments
###subsample of only STR, avr of the ones that are the same plant sp and create new df

STR_total =dat[dat[,"fungal_sp"]=="Sterile",c("plant_sp","total_biomass")]
avr_STR_total = rep(0, times=9)
plant_nam = c("C. vulgaris", "G. shallon", "K. latifolia", "P. japonica", "R. arboreum",
        "R. ferrugineum", "V. angustifolium", "V. myrtillus", "V. vitis-idaea")

for (i in 1:9){
  x = STR_total[STR_total[,"plant_sp"]==plant_nam[i],]
```

```
  avr_STR_total[i] = mean(x[,"total_biomass"], na.rm=TRUE)
}


biomass_STR_table = data.frame(plant_sp=plant_nam, biomass_STR=avr_STR_total)

###left join in dat
dat = dat%>%left_join(biomass_STR_table)

###calculate MGR = (Biomass EMF - Biomass STR)/Biomass STR in new column in the dataframe

dat$MGR = (dat$total_biomass-dat$biomass_STR)/dat$biomass_STR

################################################################################
################# RATIO ABVG/BLWG - SHIFTS IN ALLOCATION #######################
################################################################################
dat$above_below_ratio = dat$shoot_weight/dat$total_root_dry

STR_ratio =dat[dat[,"fungal_sp"]=="Sterile",c("plant_sp","above_below_ratio")]
avr_STR_ratio = rep(0, times=9)
plant_nam = c("C. vulgaris", "G. shallon", "K. latifolia", "P. japonica", "R. arboreum",
        "R. ferrugineum", "V. angustifolium", "V. myrtillus", "V. vitis-idaea")

for (i in 1:9){
  x = STR_ratio[STR_total[,"plant_sp"]==plant_nam[i],]
  avr_STR_ratio[i] = mean(x[,"above_below_ratio"], na.rm=TRUE)
}

ratio_STR_table = data.frame(plant_sp=plant_nam, ratio_STR=avr_STR_ratio)
dat = dat%>%left_join(ratio_STR_table)

###calculate MGR = (Biomass EMF - Biomass STR)/Biomass STR in new column in the dataframe

dat$MGR_ratio = (dat$above_below_ratio-dat$ratio_STR)/dat$ratio_STR
```

```
png("figures/abvg_blwg_ratio.jpg", width = 30, height = 8, units ='in', res = 300)

ggplot(data=dat, mapping=aes(x=fungal_sp, fill=fungal_sp, y=above_below_ratio)) +
 facet_grid(cols = vars(plant_sp)) +
 scale_color_brewer(palette = "Paired") +
 geom_boxplot()+
 #geom_text(aes(label=round(total_percent_colonization, digits = 2)), position=position_dodge(width =1.2), vjust=1.6, color="black", size=3.5)+
 theme(panel.grid.major = element_blank(), # get rid of major grid
    panel.grid.minor = element_blank(), # get rid of minor grid
    plot.background = element_rect(fill = "transparent",
                    colour = NA_character_), # necessary to avoid drawing plot outline
    legend.background = element_rect(fill = "transparent"),
    legend.box.background = element_rect(fill = "transparent"),
    legend.key = element_rect(fill = "transparent")) +
 ggtitle(label = "Ratio abvg/blwg") +
 theme(plot.title = element_text(hjust = 0.5)) +
 geom_hline(yintercept=0, linetype='dotted', col = "black")
dev.off()



write.csv2(dat, "Harvested_Plants_Processed_try1_updated.csv", row.names = FALSE)
```

## 2. differences_estimates.R

```
library(tidyverse)
library(ggplot2)
library(ggsci)
library(RColorBrewer)
library(lme4)
library(lmtest)
library(lmerTest)
library(sjPlot)



dat_og = read.csv2("measurements_21stFebruary_Version.csv")
```

```
str(dat_og)

dat_og = dat_og[1:714, 1:9]

dat_og =dat_og %>%select(-c("Height.t1"))

dat2 = read.csv2("Harvested_Plants_june.csv")


str(dat2)

dat2 = dat2[1:643, 1:19]

dat2 = dat2 %>% select(-c("plant_sp", "fungal_sp", "replicate", "treatment",
              "block", "Height.t0", "t1.t0", "Dead.Sick", "X.cassette"))

str(dat2)

dat = dat_og %>%
  left_join(dat2, by = "unique_id")

str(dat)

##### CHANGE PLANT SP NAMES FOR LONG NAME

dat <- transform(dat,plant_sp=gsub(pattern="CVU", replacement="C_vulgaris", plant_sp))
dat <- transform(dat,plant_sp=gsub(pattern="GSH", replacement="G_shallon", plant_sp))
dat <- transform(dat,plant_sp=gsub(pattern="KLA", replacement="K_latifolia", plant_sp))
dat <- transform(dat,plant_sp=gsub(pattern="PJA", replacement="P_japonica", plant_sp))
dat <- transform(dat,plant_sp=gsub(pattern="RAR", replacement="R_arboreum", plant_sp))
dat <- transform(dat,plant_sp=gsub(pattern="RFE", replacement="R_ferrugineum", plant_sp))
dat <- transform(dat,plant_sp=gsub(pattern="VAN", replacement="V_angustifolium", plant_sp))
dat <- transform(dat,plant_sp=gsub(pattern="VMY", replacement="V_myrtillus", plant_sp))
dat <- transform(dat,plant_sp=gsub(pattern="VVI", replacement="V_vitis-idaea", plant_sp))
```

```
##### CHANGE FUNGAL SP NAMES FOR LONG NAME

dat <- transform(dat,fungal_sp=gsub(pattern="JPK-132", replacement="Serendipitaceae_sp", fungal_sp))
dat <- transform(dat,fungal_sp=gsub(pattern="JPK-87", replacement="K_argillacea", fungal_sp))
dat <- transform(dat,fungal_sp=gsub(pattern="MEB", replacement="H_bicolor", fungal_sp))
dat <- transform(dat,fungal_sp=gsub(pattern="MEV", replacement="H_variabilis", fungal_sp))
dat <- transform(dat,fungal_sp=gsub(pattern="MGK", replacement="H_gryndleri", fungal_sp))
dat <- transform(dat,fungal_sp=gsub(pattern="OMA", replacement="O_maius", fungal_sp))
dat <- transform(dat,fungal_sp=gsub(pattern="RER", replacement="H_hepaticicola_1", fungal_sp))
dat <- transform(dat,fungal_sp=gsub(pattern="RHE", replacement="H_hepaticicola_2", fungal_sp))
dat <- transform(dat,fungal_sp=gsub(pattern="STR", replacement="Sterile", fungal_sp))


dat = dat %>% select(-c("replicate", "treatment", "block", "Height.t0",
           "Dead.Sick", "unique_id", "Height.t1", "blockx", "shoot_weight"))
####

##calculate average wet/dry biomass ratio for each plant_sp

dat1 <- data.frame()

# add real values of wet and dry weight from experiment to the table
#need to do in 2 steps: first until row 243 - where largesubsample was done

ratio_real_values <- filter(dat, random_label<=243)
ratio_real_values = ratio_real_values %>% select(-c("fungal_sp", "total_root_wet", "random_label",
                   "small_subsample_wet", "total_root_dry"))

colnames(ratio_real_values)[colnames(ratio_real_values) == c("plant_sp", "large_subsample_wet","large_subsample_dry") ] <- c("Plant.sp", "weight.wet.root", "weight.dry.root")
dat1 <- rbind(dat1, ratio_real_values)

# and then from 244 until the end with total_root_wet and total_root_dry
ratio_real_values1 <- filter(dat, random_label>243)
ratio_real_values1 = ratio_real_values1 %>% select(-c("fungal_sp", "random_label",
```

```
                                        "large_subsample_wet", "small_subsample_wet",
                                        "large_subsample_dry"))


colnames(ratio_real_values1)[colnames(ratio_real_values1) == c("plant_sp", "total_root_wet","total_root_dry") ] <- c("Plant.sp", "weight.wet.root", "weight.dry.root")

dat1 <- rbind(dat1, ratio_real_values1)


#group by

#mean (in tidyverse)

dat1 = dat1 %>% drop_na()


dat1$ratio = dat1$weight.dry.root/dat1$weight.wet.root


xx=dat1%>%

  group_by(Plant.sp)%>%

  summarize(Mean = mean(ratio))


ratio_table = data.frame(xx)

colnames(ratio_table) <- c("plant_sp", "ratio")


dat1 = dat1 %>% left_join(xx)

dat1 = dat1 %>% select(-c("ratio"))


dat1$weight.dry.estimated = dat1$weight.wet.root*dat1$Mean


dat1$dif.est = dat1$weight.dry.root-dat1$weight.dry.estimated


df <- dat1 %>% slice(-146)


library(ggpubr)


dat1$Plant.sp <- factor(dat1$Plant.sp,levels = c("G_shallon", "V_myrtillus", "P_japonica",

                                  "V_vitis-idaea", "C_vulgaris", "V_angustifolium",

                                  "K_latifolia", "R_arboreum", "R_ferrugineum"))


png("figures/difference_real_values_and_estimates_updated_all rows.jpg", width = 17, height = 3.5, units ='in', res = 300)
```

```
ggplot(data=dat1, mapping=aes(x=weight.dry.root, y=weight.dry.estimated)) +
 facet_grid(cols = vars(Plant.sp)) +
 scale_color_brewer(palette = "Paired") +
 geom_point(alpha=0.3) +
 geom_smooth(method="lm", linewidth = 0.5, alpha=0.1) +
 theme_classic(base_size = 15) +
 theme(
  panel.background = element_rect(fill = NA, color = "grey", size = 0.5),
  panel.grid.major = element_blank(),
  panel.grid.minor = element_blank(),
  plot.background = element_rect(fill = "transparent", colour = NA_character_),
  strip.background = element_rect(fill = "grey90", color = "grey", size = 0.5),
  legend.background = element_rect(fill = "transparent"),
  legend.box.background = element_rect(fill = "transparent"),
  legend.key = element_rect(fill = "transparent"),
  axis.title.x = element_text(size = 16),
  axis.line = element_line(size = 0.5),
  strip.text = element_text(size = 15),
  plot.title = element_text(hjust = 0.5, size = 16),
  axis.title.y = element_text(size = 16),
  legend.title = element_text(size = 17),
  legend.text = element_text(size = 16)
 ) +
 theme(legend.position = "none",
    panel.background = element_rect(fill = NA, color = "grey"),
    panel.spacing = unit(1, "lines"))+
 stat_cor(aes(label = after_stat(rr.label)), color = "black", geom = "label") +
 labs(y= "estimated dry weight", x = "real dry weight") +
 theme(aspect.ratio = 1.2)
dev.off()

## without outlier

df$Plant.sp <- factor(df$Plant.sp,levels = c("G_shallon", "V_myrtillus", "P_japonica",
                          "V_vitis-idaea", "C_vulgaris", "V_angustifolium",
```

```
                             "K_latifolia", "R_arboreum", "R_ferrugineum"))


png("figures/difference_real_values_and_estimates_updated.jpg", width = 17, height = 3.5, units ='in', res = 300)
ggplot(data=df, mapping=aes(x=weight.dry.root, y=weight.dry.estimated)) +
 facet_grid(cols = vars(Plant.sp)) +
 scale_color_brewer(palette = "Paired") +
 geom_point(alpha=0.3) +
 geom_smooth(method="lm", linewidth = 0.5, alpha=0.1) +
 theme_classic(base_size = 15) +
 theme(
  panel.background = element_rect(fill = NA, color = "grey", size = 0.5),
  panel.grid.major = element_blank(),
  panel.grid.minor = element_blank(),
  plot.background = element_rect(fill = "transparent", colour = NA_character_),
  strip.background = element_rect(fill = "grey90", color = "grey", size = 0.5),
  legend.background = element_rect(fill = "transparent"),
  legend.box.background = element_rect(fill = "transparent"),
  legend.key = element_rect(fill = "transparent"),
  axis.title.x = element_text(size = 16),
  axis.line = element_line(size = 0.5),
  strip.text = element_text(size = 15),
  plot.title = element_text(hjust = 0.5, size = 16),
  axis.title.y = element_text(size = 16),
  legend.title = element_text(size = 17),
  legend.text = element_text(size = 16)
 ) +
 theme(legend.position = "none",
     panel.background = element_rect(fill = NA, color = "grey"),
     panel.spacing = unit(1, "lines"))+
 stat_cor(aes(label = after_stat(rr.label)), color = "black", geom = "label") +
 labs(y= "estimated dry weight", x = "real dry weight") +
 theme(aspect.ratio = 1.2)
dev.off()
```

# 3. colonization_assessment.R

```
###### TEST FOR MODELS

library(tidyverse)

library(ggplot2)

library(RColorBrewer)

library(lme4)

library(nmixgof)

library(DHARMa)

library(emmeans)

library(glmmTMB)

library(lmtest)

library(Matrix)

library(emmeans)

library(lsmeans)

library(tidyr)

library(lmerTest)

library(ggsignif)

library(multcompView)

library(pbkrtest)

library(forcats)

library(sjPlot)

library(sjmisc)

library(dplyr)

library(stringr)


####

#homogeneity function

resid_plot_fit <- function(mod,dat) {

 E1 <- resid(mod, type = "pearson")

 N <- nrow(dat)

 p <- length(coef(dat))

 sum(E1^2)/(N-p)

 F1 <- fitted(mod)

 ggplot() +

  aes(F1, E1) +
```

```
  geom_point() +
  ggtitle("Residual plot") +
  xlab("Fitted Values") +
  ylab("Pearson residuals")
}

### COLONIZATION ASSESSMENT for coils!

# import csv with intersections + sum all intersections of each sample
col_ass = read.csv2("Colonization_Assessment1.csv")
col_ass = col_ass[,1:8]
str(col_ass)
summary(col_ass)

col_ass = col_ass %>% drop_na()

# dataframe for coils
coils_ass = col_ass %>%
 group_by(random_label) %>%
 summarize(colonized = sum(Coils),
      intersections_c = sum(roots_crossed))

coils_ass$non_colonized = coils_ass$intersections_c-coils_ass$colonized
coils_ass$percent_colonization = coils_ass$colonized/coils_ass$intersections_c
str(coils_ass)
summary(coils_ass)

#dataframe for hyphae
hyphae_ass = col_ass %>%
 group_by(random_label) %>%
 summarize(colonized_h = sum(Hyphae),
      intersections_h = sum(roots_crossed))

hyphae_ass$non_colonized_h = hyphae_ass$intersections_h-hyphae_ass$colonized_h
hyphae_ass$percent_colonization_h = hyphae_ass$colonized_h/hyphae_ass$intersections_h
```

```
str(hyphae_ass)
summary(hyphae_ass)

#dataframe for conidiophores
spikes_ass = col_ass %>%
  group_by(random_label) %>%
  summarize(colonized_s = sum(Spikes),
        intersections_s = sum(roots_crossed))

spikes_ass$non_colonized_s = spikes_ass$intersections_s-spikes_ass$colonized_s
spikes_ass$percent_colonization_s = spikes_ass$colonized_s/spikes_ass$intersections_s
str(spikes_ass)
summary(spikes_ass)

# import master spread sheet and see just colonization with coils
dat = read.csv2("Harvested_Plants_Processed_try1_updated.csv")

dat = dat %>% select(-c("replicate", "treatment", "blockx", "above_below_ratio",
            "Dead.Sick", "Height.t1", "abvg_biomass_STR", "blwg_biomass_STR",
            "total_root_wet", "small_subsample_wet", "large_subsample_wet",
            "shoot_weight", "ratio", "biomass_STR",
            "total_biomass","large_subsample_dry",
            "total_root_dry", "small_subsample_dry", "ratio_STR", "MGR_ratio"))

dat = dat%>%left_join(coils_ass)
dat = dat%>%left_join(hyphae_ass)
dat = dat%>%left_join(spikes_ass)
dat = dat %>% drop_na(percent_colonization)

#percentage of samples colonized
m = dat %>% count(percent_colonization!=0)
percentage = m[2, 2]/(m[1,2]+m[2, 2])*100

m1 = dat %>% count(percent_colonization_h!=0)
percentage1 = m1[2, 2]/(m1[1,2]+m1[2, 2])*100
```

```
m2 = dat %>% count(percent_colonization_s!=0)

percentage2 = m2[2, 2]/(m2[1,2]+m2[2, 2])*100


#################################################################

## 2. str vs plant_sp*fungal_sp


mod = glmmTMB(cbind(colonized, non_colonized) ~fungal_sp*plant_sp,
        family = binomial, data=dat)


# check assumptions

resid_plot_fit(mod,dat)

testZeroInflation(mod)

testDispersion(mod)


#save model output

# Extract fixed effects coefficients and statistics

fixed_effects <- as.data.frame(summary(mod)$coefficients$cond)


# Write to CSV

write.csv2(fixed_effects, "C:/Users/Dpao/Desktop/Master's Biology/Master Thesis/#2 semester project/Ericoid Project/colonization_model_output_5thfeb.csv", row.names=TRUE)


ref1 = emmeans::emmeans(mod, ~fungal_sp*plant_sp, type="response")

ref1

summary(ref1)


#Add p-value and statistical tests

ref1 = update(ref1, infer = c(TRUE, TRUE), null = log(35), type="response",
         calc = c(n = ".wgt."))

summary(ref1)


ref.table1 = as.data.frame(ref1)


# Write to CSV
```

```
write.csv2(ref.table1, "C:/Users/Dpao/Desktop/Master's Biology/Master Thesis/#2 semester project/Ericoid
Project/colonization_emmeans_output_5thfeb.csv", row.names=TRUE)

ref.table1$fungal_sp <- gsub(fixed("Sterile"), "Non-inoculated", ref.table1$fungal_sp)

ref.table1$plant_sp <- factor(ref.table1$plant_sp,levels = c("G. shallon", "V. myrtillus", "P. japonica",
                          "V. vitis-idaea", "C. vulgaris", "V. angustifolium",
                          "K. latifolia", "R. arboreum", "R. ferrugineum"))

# Create color palette with ordered species
library(RColorBrewer)

# colors_definition.R
fungal_colors <- c(
  "Non-inoculated" = "#66C2A5",
  "H. gryndleri" = "#FC8D62",
  "H. hepaticicola_PK 135-3" = "#8DA0CB",
  "H. hepaticicola_UAMH7357/ICMP18553" = "#E78AC3",
  "K. argillacea" = "#A6D854",
  "H. bicolor" = "#FFD92F",
  "H. variabilis" = "#E5C494",
  "O. maius" = "#B3B3B3",
  "Serendipitaceae_sp" = "#7570B3"
)
saveRDS(fungal_colors, "fungal_colors.rds")

ref.table1$fungal_sp <- factor(ref.table1$fungal_sp,
                levels = c("Non-inoculated", "H. gryndleri", "H. hepaticicola_PK 135-3", "H. hepaticicola_UAMH7357/ICMP18553",
                   "K. argillacea", "H. bicolor", "H. variabilis",
                   "O. maius", "Serendipitaceae_sp"))

png("figures/colonization/col_str_treatment_update.jpg", width = 18, height = 5.4, units ='in', res = 300)
  ggplot(ref.table1, aes(fungal_sp, prob, color = fungal_sp, fill = fungal_sp)) +
  facet_grid(cols = vars(plant_sp)) +
  geom_bar(stat="identity", alpha = 0.08, width=0.8) +
  geom_point(size = 1.2) +
```

```
  geom_errorbar(aes(ymin=prob-asymp.LCL, ymax=prob+asymp.UCL), width=.65, linewidth=0.6, position=position_dodge(1)) +
  scale_fill_manual(values = fungal_colors) +
  scale_color_manual(values = fungal_colors) +
   theme_classic(base_size = 15) +
   theme(
     panel.background = element_rect(fill = NA, color = "grey", size = 0.5),
     panel.grid.major = element_blank(),
     panel.grid.minor = element_blank(),
     plot.background = element_rect(fill = "transparent", colour = NA_character_),
     strip.background = element_rect(fill = "grey90", color = "grey", size = 0.5),
     legend.background = element_rect(fill = "transparent"),
     legend.box.background = element_rect(fill = "transparent"),
     legend.key = element_rect(fill = "transparent"),
     axis.title.x = element_blank(),
     axis.text.x = element_blank(),
     axis.ticks.x = element_blank(),
     axis.line = element_line(size = 0.5),
     strip.text = element_text(size = 14),
     plot.title = element_text(hjust = 0.5, size = 16),
     axis.title.y = element_text(size = 16),
     legend.title = element_text(size = 14),
     legend.text = element_text(size = 13)
   ) +
  ylab("Root colonization rate") +
  geom_hline(yintercept=0, linetype='dotted', col = "black", size=0.1)
dev.off()

tab_results = pairs(ref1)

makeStars <- function(x){
  stars <- c("****", "***", "**", "*", "ns")
  vec <- c(0, 0.0001, 0.001, 0.01, 0.05, 1.01)
  i <- findInterval(x, vec)
  stars[i]
}
```

```
tab_results = as.data.frame(tab_results)


tab_results = separate(tab_results, col=contrast, into=c('comb1', 'comb2'), sep=' / ')

tab_results$p_value_stars <- makeStars(tab_results$p.value)


#remove "(" and ")" from some of the cells

tab_results$comb1 <- gsub("\\(", "", tab_results$comb1)

tab_results$comb2 <- gsub("\\(", "", tab_results$comb2)

tab_results$comb1 <- gsub("\\)", "", tab_results$comb1)

tab_results$comb2 <- gsub("\\)", "", tab_results$comb2)


#separate comb1 into fungi1 and plant1 and comb2 into fungi2 plant2

# split the combination based on the plant name pattern

split_combination <- function(combination) {

 # Create a pattern with all possible plant names

 # Escape special characters like "-" and "."

 plant_patterns <- c(

  "G\\. shallon", "V\\. myrtillus", "P\\. japonica",

  "V\\. vitis-idaea", "C\\. vulgaris", "V\\. angustifolium",

  "K\\. latifolia", "R\\. arboreum", "R\\. ferrugineum"

 )


 # Create a regex pattern that matches any of the plant names

 plant_pattern <- paste0("(", paste(plant_patterns, collapse="|"), ")$")


 # Extract the plant name

 plant_name <- str_extract(combination, plant_pattern)


 # Extract the fungi name by removing the plant name

 fungi_name <- str_replace(combination, paste0(" ", plant_pattern), "")


 return(list(fungi = fungi_name, plant = plant_name))

}


tab_results = tab_results %>%
```

```
  mutate(
    comb1_split = map(comb1, split_combination),
    comb2_split = map(comb2, split_combination),

    fungi1 = map_chr(comb1_split, "fungi"),
    plant1 = map_chr(comb1_split, "plant"),
    fungi2 = map_chr(comb2_split, "fungi"),
    plant2 = map_chr(comb2_split, "plant")
  ) %>%
  select(-comb1_split, -comb2_split)  # Remove the intermediate columns

tab_results = tab_results %>%
  select(-comb1, -comb2)

# keep rows with “sterile” and plant1=plant2
filtered_results <- tab_results[
  (tab_results$fungi1 == "Sterile" | tab_results$fungi2 == "Sterile") &
    (tab_results$plant1 == tab_results$plant2),
]

filtered_results <- filtered_results[order(filtered_results$plant1), ]
summary_table <- table(filtered_results$plant1)
print(summary_table)

write.csv2(filtered_results, "C:/Users/Dpao/Desktop/Master's Biology/Master Thesis/#2 semester project/Ericoid Project/colonization_pairs_3rdmar.csv", row.names=FALSE)

##### colonization and MGR
# make categorical
dat$fungal_sp <- to_factor(dat$fungal_sp)
dat$plant_sp <- to_factor(dat$plant_sp)

dat1 = subset(dat, dat[,"fungal_sp"]!="Sterile")
dat1 = dat1 %>% drop_na(MGR)

m = min(dat1$MGR)
```

```
n = m*-1+0.01

#tried with:
colm = lmer(log(MGR+n)~plant_sp*percent_colonization*fungal_sp +   Height.t0 + (1|block), data=dat1)
colm1 = lmer(log(MGR+n)~plant_sp*percent_colonization + fungal_sp*percent_colonization +
        plant_sp*fungal_sp + Height.t0 + (1|block), data=dat1)

colm2 = lmer(log(MGR+n)~fungal_sp*percent_colonization + plant_sp + Height.t0 + (1|block), data=dat1)
colm3 = lmer(log(MGR+n)~plant_sp*percent_colonization + fungal_sp + Height.t0 + (1|block), data=dat1)

colm4 = lmer(log(MGR+n)~plant_sp + percent_colonization + fungal_sp + Height.t0 + (1|block), data=dat1)

summary(colm4)
a = anova(colm4)
plot(colm4)

#aboveground
o = min(dat1$MGR_abvg)
p = o*-1+0.01
colm = lmer(log(MGR_abvg+p)~plant_sp + percent_colonization + fungal_sp + Height.t0 + (1|block), data=dat1)
summary(colm)
b = anova(colm)
plot(colm)

#belowground
q = min(dat1$MGR_blwg)
r = q*-1+0.01
colmx = lmer(log(MGR_blwg+r)~plant_sp + percent_colonization + fungal_sp + Height.t0 + (1|block), data=dat1)
summary(colmx)
c = anova(colmx)
plot(colmx)

write.csv2(a, "C:/Users/Dpao/Desktop/Master's Biology/Master Thesis/#2 semester project/Ericoid Project/MGRtotal_col_updated.csv", row.names=TRUE)

write.csv2(b, "C:/Users/Dpao/Desktop/Master's Biology/Master Thesis/#2 semester project/Ericoid Project/MGRabvg_col_updated.csv", row.names=TRUE)
```

```
write.csv2(c, "C:/Users/Dpao/Desktop/Master's Biology/Master Thesis/#2 semester project/Ericoid Project/MGRblwg_col_updated.csv", row.names=TRUE)


#### colonization and MGR (hyphal coils) - subset of plant-fungi combinations

## that had significantly higher colonization than control

#from filtered_results take combinations that are not significantly different

combinations_contaminated = filtered_results %>%

  select (-c(odds.ratio, SE, df, null, z.ratio, p.value, fungi2, plant2))


combinations_contaminated <- combinations_contaminated %>%

  filter(!(p_value_stars!= "ns")) %>%

  rename(fungal_sp = fungi1, plant_sp = plant1) %>%

  select(-c(p_value_stars))


###from dat1 remove the plant-fungi combinations that are present in combinations_contaminated

dat_subset <- dat1 %>%

  anti_join(combinations_contaminated, by = c("plant_sp", "fungal_sp"))


ms = min(dat_subset$MGR)

ns = ms*-1+0.01


#tried with:

colms = lmer(log(MGR+ns)~plant_sp*percent_colonization*fungal_sp +   Height.t0 + (1|block), data=dat_subset)

colm1s = lmer(log(MGR+ns)~plant_sp*percent_colonization + fungal_sp*percent_colonization +

        plant_sp*fungal_sp + Height.t0 + (1|block), data=dat_subset)


colm2s = lmer(log(MGR+ns)~fungal_sp*percent_colonization + plant_sp + Height.t0 + (1|block), data=dat_subset)

colm3s = lmer(log(MGR+ns)~plant_sp*percent_colonization + fungal_sp + Height.t0 + (1|block), data=dat_subset)


colm4s = lmer(log(MGR+ns)~plant_sp + percent_colonization + fungal_sp + Height.t0 + (1|block), data=dat_subset)


summary(colm4s)

as = anova(colm4s)

plot(colm4s)


#aboveground
```

```
os = min(dat_subset$MGR_abvg)

ps = os*-1+0.01

colms = lmer(log(MGR_abvg+ps)~plant_sp + percent_colonization + fungal_sp + Height.t0 + (1|block), data=dat_subset)

summary(colms)

bs = anova(colms)

plot(colms)


#belowground

qs = min(dat_subset$MGR_blwg)

rs = qs*-1+0.01

colmxs = lmer(log(MGR_blwg+rs)~plant_sp + percent_colonization + fungal_sp + Height.t0 + (1|block), data=dat_subset)

summary(colmxs)

cs = anova(colmxs)

plot(colmxs)



write.csv2(as, "C:/Users/Dpao/Desktop/Master's Biology/Master Thesis/#2 semester project/Ericoid Project/MGRtotal_col_subset.csv", row.names=TRUE)

write.csv2(bs, "C:/Users/Dpao/Desktop/Master's Biology/Master Thesis/#2 semester project/Ericoid Project/MGRabvg_col_subset.csv", row.names=TRUE)

write.csv2(cs, "C:/Users/Dpao/Desktop/Master's Biology/Master Thesis/#2 semester project/Ericoid Project/MGRblwg_col_subset.csv", row.names=TRUE)


##### colonization and MGR - hyphae

#total biomass

m = min(dat1$MGR)

n = m*-1+0.01


colm_h1 = lmer(log(MGR+n)~plant_sp + percent_colonization_h + fungal_sp + Height.t0 + (1|block), data=dat1)


summary(colm_h1)

d = anova(colm_h1)

plot(colm_h1)


#aboveground

o = min(dat1$MGR_abvg)
```

```
p = o*-1+0.01

colm_h2 = lmer(log(MGR_abvg+p)~plant_sp + percent_colonization_h + fungal_sp + Height.t0 + (1|block), data=dat1)

summary(colm_h2)

e = anova(colm_h2)

plot(colm_h2)


#belowground

q = min(dat1$MGR_blwg)

r = q*-1+0.01

colm_h3 = lmer(log(MGR_blwg+r)~plant_sp + percent_colonization_h + fungal_sp + Height.t0 + (1|block), data=dat1)

summary(colm_h3)

f = anova(colm_h3)

plot(colm_h3)


write.csv2(d, "C:/Users/Dpao/Desktop/Master's Biology/Master Thesis/#2 semester project/Ericoid Project/MGRtotal_hyphae_updated.csv", row.names=TRUE)

write.csv2(e, "C:/Users/Dpao/Desktop/Master's Biology/Master Thesis/#2 semester project/Ericoid Project/MGRabvg_hyphae_updated.csv", row.names=TRUE)

write.csv2(f, "C:/Users/Dpao/Desktop/Master's Biology/Master Thesis/#2 semester project/Ericoid Project/MGRblwg_hyphae_updated.csv", row.names=TRUE)


##### colonization and MGR - conidiophores

#total biomass

m = min(dat1$MGR)

n = m*-1+0.01


colm_c1 = lmer(log(MGR+n)~plant_sp + percent_colonization_s + fungal_sp + Height.t0 + (1|block), data=dat1)


summary(colm_c1)

g = anova(colm_c1)

plot(colm_c1)


#aboveground

o = min(dat1$MGR_abvg)

p = o*-1+0.01

colm_c2 = lmer(log(MGR_abvg+p)~plant_sp + percent_colonization_s + fungal_sp + Height.t0 + (1|block), data=dat1)
```

```
summary(colm_c2)

h = anova(colm_c2)

plot(colm_c2)


#belowground

q = min(dat1$MGR_blwg)

r = q*-1+0.01

colm_c3 = lmer(log(MGR_blwg+r)~plant_sp + percent_colonization_s + fungal_sp + Height.t0 + (1|block), data=dat1)

summary(colm_c3)

i = anova(colm_c3)

plot(colm_c3)


write.csv2(g, "C:/Users/Dpao/Desktop/Master's Biology/Master Thesis/#2 semester project/Ericoid Project/MGRtotal_conidiophores_updated.csv", row.names=TRUE)

write.csv2(h, "C:/Users/Dpao/Desktop/Master's Biology/Master Thesis/#2 semester project/Ericoid Project/MGRabvg_conidiophores_updated.csv", row.names=TRUE)

write.csv2(i, "C:/Users/Dpao/Desktop/Master's Biology/Master Thesis/#2 semester project/Ericoid Project/MGRblwg_conidiophores_updated.csv", row.names=TRUE)


######################################################################

######################### HYPHAE & SPIKES ############################

######################################################################

# models didn't work, so plot real values in boxplot

################################################################

dat$fungal_sp <- gsub(fixed("Sterile"), "Non-inoculated", dat$fungal_sp)


dat$plant_sp <- factor(dat$plant_sp,levels = c("G. shallon", "V. myrtillus", "P. japonica",

                                "V. vitis-idaea", "C. vulgaris", "V. angustifolium",

                                "K. latifolia", "R. arboreum", "R. ferrugineum"))


dat$fungal_sp <- factor(dat$fungal_sp,levels = c("Non-inoculated", "H. gryndleri", "H. hepaticicola_PK 135-3", "H. hepaticicola_UAMH7357/ICMP18553",

                                 "K. argillacea", "H. bicolor", "H. variabilis",

                                 "O. maius", "Serendipitaceae_sp"))


#plot hyphal colonization

png("figures/colonization/hyphae_col_assessment_updated.jpg", width = 18, height = 5.4, units ='in', res = 300)
```

```
ggplot(dat, aes(fungal_sp, percent_colonization_h, color = fungal_sp, fill = fungal_sp)) +
 facet_grid(cols = vars(plant_sp)) +
 geom_boxplot(alpha = 0.1, width=0.4) +
 scale_fill_manual(values = fungal_colors) +
 scale_color_manual(values = fungal_colors) +
 theme_classic(base_size = 15) +
 theme(
  panel.background = element_rect(fill = NA, color = "grey", size = 0.5),
  panel.grid.major = element_blank(),
  panel.grid.minor = element_blank(),
  plot.background = element_rect(fill = "transparent", colour = NA_character_),
  strip.background = element_rect(fill = "grey90", color = "grey", size = 0.5),
  legend.background = element_rect(fill = "transparent"),
  legend.box.background = element_rect(fill = "transparent"),
  legend.key = element_rect(fill = "transparent"),
  axis.title.x = element_blank(),
  axis.text.x = element_blank(),
  axis.ticks.x = element_blank(),
  axis.line = element_line(size = 0.5),
  strip.text = element_text(size = 14),
  plot.title = element_text(hjust = 0.5, size = 16),
  axis.title.y = element_text(size = 16),
  legend.title = element_text(size = 14),
  legend.text = element_text(size = 13)
 ) +
 ylab("Root colonization rate - Hyphae") +
 theme(axis.title.x=element_blank())
dev.off()

#plot conidiophores
png("figures/colonization/conidiophores_col_assessment_updated.jpg", width = 18, height = 5.4, units ='in', res = 300)
ggplot(dat, aes(fungal_sp, percent_colonization_s, color = fungal_sp, fill = fungal_sp)) +
 facet_grid(cols = vars(plant_sp)) +
 geom_boxplot(alpha = 0.1, width=0.4) +
 scale_fill_manual(values = fungal_colors) +
```

```
  scale_color_manual(values = fungal_colors) +
  theme_classic(base_size = 15) +
  theme(
    panel.background = element_rect(fill = NA, color = "grey", size = 0.5),
    panel.grid.major = element_blank(),
    panel.grid.minor = element_blank(),
    plot.background = element_rect(fill = "transparent", colour = NA_character_),
    strip.background = element_rect(fill = "grey90", color = "grey", size = 0.5),
    legend.background = element_rect(fill = "transparent"),
    legend.box.background = element_rect(fill = "transparent"),
    legend.key = element_rect(fill = "transparent"),
    axis.title.x = element_blank(),
    axis.text.x = element_blank(),
    axis.ticks.x = element_blank(),
    axis.line = element_line(size = 0.5),
    strip.text = element_text(size = 14),
    plot.title = element_text(hjust = 0.5, size = 16),
    axis.title.y = element_text(size = 16),
    legend.title = element_text(size = 14),
    legend.text = element_text(size = 13)
  ) +
  ylab("Root colonization rate - Conidiophores") +
  theme(axis.title.x=element_blank())
dev.off()
```

## 4. plant_models_MGR.R

```
library(tidyverse)
library(lme4)
library(lmtest)
library(Matrix)
library(emmeans)
library(ggplot2)
library(lsmeans)
library(tidyr)
library(lmerTest)
```

```
library(ggsignif)
library(multcompView)
library(pbkrtest)
library(forcats)
library(egg)

###############################################################################
########### FUNCTIONS NEEDED IN THIS SCRIPT
# Load color data
fungal_colors <- readRDS("fungal_colors.rds")

#make stars for p-value
makeStars <- function(x){
  stars <- c("****", "***", "**", "*", "ns")
  vec <- c(0, 0.0001, 0.001, 0.01, 0.05, 1.01)
  i <- findInterval(x, vec)
  stars[i]
}
###############################################################################
##using measurements.csv - height of sick-looking plants was deleted
######to give the correct number of samples in the plots
#read data

dat = read.csv2("Harvested_Plants_Processed_try1_updated.csv")
str(dat)

dat$plant_sp=as.factor(dat$plant_sp)
dat$fungal_sp=as.factor(dat$fungal_sp)

summary(dat)
str(dat)

#removing columns from master spreadsheet that are not important here
# keeping:
dat = dat%>%
```

```
  select(-c("Dead.Sick", "Height.t1", "blockx", "total_root_wet", "small_subsample_wet",
         "large_subsample_wet", "large_subsample_dry", "shoot_weight",
         "ratio", "small_subsample_dry", "total_root_dry", "abvg_biomass_STR",
         "blwg_biomass_STR", "total_biomass", "biomass_STR", "ratio_STR"))
str(dat)
summary(dat)
dat = dat %>% drop_na()

summary(dat)
str(dat)

#table w/out STR treatment
dat1 = subset(dat, dat[,"fungal_sp"]!="Sterile")

##############################################################################
#############################ABOVEGROUND######################################
##############################################################################
#1. model
#2. emmeans with plant*fungi interaction and only plant
#3. plot MGR
#4. pairwise comparison
################ model ################
m = min(dat1$MGR_abvg)
n = m*-1+0.01

bm1 = lmer(log(MGR_abvg+n)~fungal_sp * plant_sp + Height.t0 + (1|block), data=dat1)
summary(bm1)
anova(bm1)
plot(bm1)

#save model output, extract fixed effects coefficients and statistics
abvg_model_output <- as.data.frame(summary(bm1)$coefficients)

# Write to CSV - model output
write.csv2(abvg_model_output, "C:/Users/Dpao/Desktop/Master's Biology/Master Thesis/#2 semester project/Ericoid Project/MGR_abvg_model_output_5thjfeb.csv", row.names=TRUE)
```

```
############# emmeans ###############

means = emmeans::emmeans(bm1, ~fungal_sp*plant_sp, type="response")

means

summary(means)


#Add p-value and statistical tests

means = update(means, infer = c(TRUE, TRUE), null = log(35), type="response",

            calc = c(n = ".wgt."))

summary(means)


meas_abvg = summary(means)

meas_abvg$response


# Write to CSV - emmeans output

write.csv2(meas_abvg, "C:/Users/Dpao/Desktop/Master's Biology/Master Thesis/#2 semester project/Ericoid Project/MGR_abvg_emmeans_output_5thjfeb.csv", row.names=TRUE)


## emmeans for plant only

means_abvg_p = emmeans::emmeans(bm1, ~plant_sp, type="response")


means_abvg_p

summary(means_abvg_p)


#Add p-value and statistical tests

means_abvg_p = update(means_abvg_p, infer = c(TRUE, TRUE), null = log(35), type="response",

                  calc = c(n = ".wgt."))

summary(means_abvg_p)


meas_abvg_p = summary(means_abvg_p)


# Write to CSV - emmeans plants across fungal treatment

write.csv2(meas_abvg_p, "C:/Users/Dpao/Desktop/Master's Biology/Master Thesis/#2 semester project/Ericoid Project/MGR_abvg_emmeans_plants_output_5thfeb.csv", row.names=TRUE)


## emmeans for fungi only
```

```
means_abvg_f = emmeans::emmeans(bm1, ~fungal_sp, type="response")


means_abvg_f

summary(means_abvg_f)


#Add p-value and statistical tests

means_abvg_f = update(means_abvg_f, infer = c(TRUE, TRUE), null = log(35), type="response",

            calc = c(n = ".wgt."))

summary(means_abvg_f)


means_abvg_f = summary(means_abvg_f)


# Write to CSV

write.csv2(means_abvg_f, "C:/Users/Dpao/Desktop/Master's Biology/Master Thesis/#2 semester project/Ericoid Project/MGR_abvg_emmeans_fungi_output_5thfeb.csv", row.names=TRUE)


###################### Plot ABOVEGROUND #########################

#add column to specify if error bars are going to be dashed or not

meas_abvg$lines=ifelse(sign(meas_abvg$lower.CL)==sign(meas_abvg$upper.CL),"1","2")


meas_abvg$plant_sp <- factor(meas_abvg$plant_sp,levels = c("G. shallon", "V. myrtillus", "P. japonica",

                         "V. vitis-idaea", "C. vulgaris", "V. angustifolium",

                         "K. latifolia", "R. arboreum", "R. ferrugineum"))


#Add Sterile Treatment to Dataset to have the empty space to make colonization and MGR plots match

# add "Sterile" to fungal species factor levels

meas_abvg$fungal_sp <- factor(meas_abvg$fungal_sp,

              levels = c(levels(meas_abvg$fungal_sp), "Non-inoculated"))


# data frame with all plant species and the Sterile treatment

sterile_rows <- data.frame(

 plant_sp = levels(meas_abvg$plant_sp),

 fungal_sp = rep("Non-inoculated", length(levels(meas_abvg$plant_sp)))

)


# Add any other necessary columns with NA or appropriate default values
```

```
# Assuming other columns exist in meas_total, add them to sterile_rows with NA
other_cols <- setdiff(names(meas_abvg), c("plant_sp", "fungal_sp"))
for(col in other_cols) {
  sterile_rows[[col]] <- 0
}

# Combine the original data with the new sterile rows
meas_abvg_with_sterile <- rbind(meas_abvg, sterile_rows)

meas_abvg_with_sterile$fungal_sp <- factor(meas_abvg_with_sterile$fungal_sp,
                              levels = c("Non-inoculated","H. gryndleri", "H. hepaticicola_PK 135-3", "H.
hepaticicola_UAMH7357/ICMP18553",
                                   "K. argillacea", "H. bicolor", "H. variabilis",
                                   "O. maius", "Serendipitaceae_sp"))


png("figures/abvg_MGR/MGR_abvg_emmeans_updatedtryout.jpg", width = 18, height = 5.4, units ='in', res = 300)
ggplot(data=meas_abvg_with_sterile, mapping=aes(x=fungal_sp, color=fungal_sp)) +
  facet_grid(cols = vars(plant_sp)) +
  # Split geom_point into two layers - one for Sterile and one for other treatments
  geom_point(data = subset(meas_abvg_with_sterile, fungal_sp == "Non-inoculated"),
        aes(x= fungal_sp, y=response),
        position=position_dodge(width =1),
        size=1.5,
        alpha=0) +
  geom_point(data = subset(meas_abvg_with_sterile, fungal_sp != "Non-inoculated"),
        aes(x= fungal_sp, y=response),
        position=position_dodge(width =1),
        size=1.5) +
  geom_errorbar(data = subset(meas_abvg_with_sterile, fungal_sp == "Non-inoculated"),
          aes(ymin=lower.CL, ymax=upper.CL),
          position=position_dodge(width =1),
          width=.75,
          linewidth=0.7,
          alpha=0) +
  geom_errorbar(data = subset(meas_abvg_with_sterile, fungal_sp != "Non-inoculated"),
```

```
            aes(ymin=lower.CL, ymax=upper.CL, linetype=lines),
            position=position_dodge(width =1),
            width=.75,
            linewidth=0.7) +
  scale_color_manual(values = fungal_colors) +
  theme_classic(base_size = 15) +
  theme(
   panel.background = element_rect(fill = NA, color = "grey", size = 0.5),
   panel.grid.major = element_blank(),
   panel.grid.minor = element_blank(),
   plot.background = element_rect(fill = "transparent", colour = NA_character_),
   strip.background = element_rect(fill = "grey90", color = "grey", size = 0.5),
   legend.background = element_rect(fill = "transparent"),
   legend.box.background = element_rect(fill = "transparent"),
   legend.key = element_rect(fill = "transparent"),
   axis.title.x = element_blank(),
   axis.text.x = element_blank(),
   axis.ticks.x = element_blank(),
   axis.line = element_line(size = 0.5),
   strip.text = element_text(size = 14),
   plot.title = element_text(hjust = 0.5, size = 16),
   axis.title.y = element_text(size = 16),
   legend.title = element_text(size = 14),
   legend.text = element_text(size = 13)
  ) +
  ylab("Mycorrhizal Growth Response") +
  ylim (-1.5, 25) +
  guides(linetype = "none") +
  labs(color = "Fungal Isolates") +
  geom_hline(yintercept=0, linetype='dotted', col = "black")
dev.off()


################## pairwise comparison #######################
tab_results = pairs(means)
```

```
tab_results = as.data.frame(tab_results)

tab_results = separate(tab_results, col=contrast, into=c('comb1', 'comb2'), sep=' - ')

tab_results$p_value_stars <- makeStars(tab_results$p.value)

#remove "(" and ")" from some of the cells
tab_results$comb1 <- gsub("\\(", "", tab_results$comb1)
tab_results$comb2 <- gsub("\\(", "", tab_results$comb2)
tab_results$comb1 <- gsub("\\)", "", tab_results$comb1)
tab_results$comb2 <- gsub("\\)", "", tab_results$comb2)

#separate comb1 into fungi1 and plant1 and comb2 into fungi2 plant2
# function that splits the combination based on the plant name pattern
split_combination <- function(combination) {
  # Create a pattern with all possible plant names
  # Escape special characters like "-" and "."
  plant_patterns <- c(
    "G\\. shallon", "V\\. myrtillus", "P\\. japonica",
    "V\\. vitis-idaea", "C\\. vulgaris", "V\\. angustifolium",
    "K\\. latifolia", "R\\. arboreum", "R\\. ferrugineum"
  )

  # Create a regex pattern that matches any of the plant names
  plant_pattern <- paste0("(", paste(plant_patterns, collapse="|"), ")$")

  # Extract the plant name
  plant_name <- str_extract(combination, plant_pattern)

  # Extract the fungi name by removing the plant name
  fungi_name <- str_replace(combination, paste0(" ", plant_pattern), "")

  return(list(fungi = fungi_name, plant = plant_name))
}
```

```
tab_results = tab_results %>%
  mutate(
    comb1_split = map(comb1, split_combination),
    comb2_split = map(comb2, split_combination),

    fungi1 = map_chr(comb1_split, "fungi"),
    plant1 = map_chr(comb1_split, "plant"),
    fungi2 = map_chr(comb2_split, "fungi"),
    plant2 = map_chr(comb2_split, "plant")
  ) %>%
  select(-comb1_split, -comb2_split)  # Remove the intermediate columns

tab_results = tab_results %>%
  select(-comb1, -comb2)

# keep only rows plant1=plant2
filtered_results_abvg <- tab_results[tab_results$plant1 == tab_results$plant2, ]

write.csv2(filtered_results_abvg, "C:/Users/Dpao/Desktop/Master's Biology/Master Thesis/#2 semester project/Ericoid Project/MGR_abvg_pairs_3rdmar.csv", row.names=FALSE)

###############################################################################
############################# BELOWGROUND ######################################
###############################################################################
#1. model
#2. emmeans with plant*fungi interaction and only plant
#3. plot MGR
#4. pairwise comparison
####################### model #########################
o = min(dat1$MGR_blwg)
p = o*-1+0.01

bm2 = lmer(log(MGR_blwg+p)~fungal_sp * plant_sp + Height.t0 + (1|block), data=dat1)
summary(bm2)
anova(bm2)
plot(bm2)
```

```
#save model output

# Extract fixed effects coefficients and statistics

blwg_model_output <- as.data.frame(summary(bm2)$coefficients)


# Write to CSV - model output

write.csv2(blwg_model_output, "C:/Users/Dpao/Desktop/Master's Biology/Master Thesis/#2 semester project/Ericoid Project/MGR_blwg_model_output_5thfeb.csv", row.names=TRUE)


########################### emmeans ###################################

means_blwg = emmeans::emmeans(bm2, ~fungal_sp*plant_sp, type="response")


means_blwg

summary(means_blwg)


#Add p-value and statistical tests

means_blwg = update(means_blwg, infer = c(TRUE, TRUE), null = log(35), type="response",

        calc = c(n = ".wgt."))

summary(means_blwg)


meas_blwg = summary(means_blwg)

meas_blwg$response


# Write to CSV - emmeans output

write.csv2(meas_blwg, "C:/Users/Dpao/Desktop/Master's Biology/Master Thesis/#2 semester project/Ericoid Project/MGR_blwg_emmeans_output_5thfeb.csv", row.names=TRUE)


## emmeans for plant only

means_blwg_p = emmeans::emmeans(bm2, ~plant_sp, type="response")


means_blwg_p

summary(means_blwg_p)


#Add p-value and statistical tests

means_blwg_p = update(means_blwg_p, infer = c(TRUE, TRUE), null = log(35), type="response",

          calc = c(n = ".wgt."))
```

```
summary(means_blwg_p)


meas_blwg_p = summary(means_blwg_p)


# Write to CSV - emmeans plants across fungal treatment
write.csv2(meas_blwg_p, "C:/Users/Dpao/Desktop/Master's Biology/Master Thesis/#2 semester project/Ericoid Project/MGR_blwg_emmeans_plants_output_5thfeb.csv", row.names=TRUE)


## emmeans for fungi only
means_blwg_f = emmeans::emmeans(bm2, ~fungal_sp, type="response")


means_blwg_f
summary(means_blwg_f)


#Add p-value and statistical tests
means_blwg_f = update(means_blwg_f, infer = c(TRUE, TRUE), null = log(35), type="response",
         calc = c(n = ".wgt."))
summary(means_blwg_f)


means_blwg_f = summary(means_blwg_f)


# Write to CSV - emmeans fungi across plant species
write.csv2(means_blwg_f, "C:/Users/Dpao/Desktop/Master's Biology/Master Thesis/#2 semester project/Ericoid Project/MGR_blwg_emmeans_fungi_output_5thfeb.csv", row.names=TRUE)


################################ Plot BELOWGROUND ########################################
#add column to specify if error bars are going to be dashed or not
meas_blwg$lines=ifelse(sign(meas_blwg$lower.CL)==sign(meas_blwg$upper.CL),"1","2")


meas_blwg$plant_sp <- factor(meas_blwg$plant_sp,levels = c("G. shallon", "V. myrtillus", "P. japonica",
                      "V. vitis-idaea", "C. vulgaris", "V. angustifolium",
                      "K. latifolia", "R. arboreum", "R. ferrugineum"))


#Add Sterile Treatment to Dataset – same reason as aboveground
meas_blwg$fungal_sp <- factor(meas_blwg$fungal_sp,
           levels = c(levels(meas_blwg$fungal_sp), "Non-inoculated"))
```

```
# Create a data frame with all plant species and the Sterile treatment
sterile_rows <- data.frame(
  plant_sp = levels(meas_blwg$plant_sp),
  fungal_sp = rep("Non-inoculated", length(levels(meas_blwg$plant_sp)))
)

# Add any other necessary columns with NA or appropriate default values
# Assuming other columns exist in meas_total, add them to sterile_rows with NA
other_cols <- setdiff(names(meas_blwg), c("plant_sp", "fungal_sp"))
for(col in other_cols) {
  sterile_rows[[col]] <- 0
}

# Combine the original data with the new sterile rows
meas_blwg_with_sterile <- rbind(meas_blwg, sterile_rows)

meas_blwg_with_sterile$fungal_sp <- factor(meas_blwg_with_sterile$fungal_sp,
                              levels = c("Non-inoculated","H. gryndleri", "H. hepaticicola_PK 135-3", "H. hepaticicola_UAMH7357/ICMP18553",
                                     "K. argillacea", "H. bicolor", "H. variabilis",
                                     "O. maius", "Serendipitaceae_sp"))

png("figures/blwg_MGR/MGR_blwg_emmeans_updatedtryout.jpg", width = 18, height = 5.4, units ='in', res = 300)
ggplot(data=meas_blwg_with_sterile, mapping=aes(x=fungal_sp, color=fungal_sp)) +
  facet_grid(cols = vars(plant_sp)) +
  # Split geom_point into two layers - one for Sterile and one for other treatments
  geom_point(data = subset(meas_blwg_with_sterile, fungal_sp == "Non-inoculated"),
        aes(x= fungal_sp, y=response),
        position=position_dodge(width =1),
        size=1.5,
        alpha=0) +
  geom_point(data = subset(meas_blwg_with_sterile, fungal_sp != "Non-inoculated"),
        aes(x= fungal_sp, y=response),
        position=position_dodge(width =1),
        size=1.5) +
```

```
geom_errorbar(data = subset(meas_blwg_with_sterile, fungal_sp == "Non-inoculated"),
          aes(ymin=lower.CL, ymax=upper.CL),
          position=position_dodge(width =1),
          width=.75,
          linewidth=0.7,
          alpha=0) +
geom_errorbar(data = subset(meas_blwg_with_sterile, fungal_sp != "Non-inoculated"),
          aes(ymin=lower.CL, ymax=upper.CL, linetype=lines),
          position=position_dodge(width =1),
          width=.75,
          linewidth=0.7) +
scale_color_manual(values = fungal_colors) +
theme_classic(base_size = 15) +
theme(
  panel.background = element_rect(fill = NA, color = "grey", size = 0.5),
  panel.grid.major = element_blank(),
  panel.grid.minor = element_blank(),
  plot.background = element_rect(fill = "transparent", colour = NA_character_),
  strip.background = element_rect(fill = "grey90", color = "grey", size = 0.5),
  legend.background = element_rect(fill = "transparent"),
  legend.box.background = element_rect(fill = "transparent"),
  legend.key = element_rect(fill = "transparent"),
  axis.title.x = element_blank(),
  axis.text.x = element_blank(),
  axis.ticks.x = element_blank(),
  axis.line = element_line(size = 0.5),
  strip.text = element_text(size = 14),
  plot.title = element_text(hjust = 0.5, size = 16),
  axis.title.y = element_text(size = 16),
  legend.title = element_text(size = 14),
  legend.text = element_text(size = 13)
) +
ylab("Mycorrhizal Growth Response") +
ylim (-1.5, 35.5) +
guides(linetype = "none") +
```

```
 labs(color = "Fungal Isolates") +
 geom_hline(yintercept=0, linetype='dotted', col = "black")
dev.off()

######################## pairwise comparisons ############################3
tab_results_b = pairs(means_blwg)
tab_results_b = as.data.frame(tab_results_b)

tab_results_b = separate(tab_results_b, col=contrast, into=c('comb1', 'comb2'), sep=' - ')

tab_results_b$p_value_stars <- makeStars(tab_results_b$p.value)

#remove "(" and ")" from some of the cells
tab_results_b$comb1 <- gsub("\\(", "", tab_results_b$comb1)
tab_results_b$comb2 <- gsub("\\(", "", tab_results_b$comb2)
tab_results_b$comb1 <- gsub("\\)", "", tab_results_b$comb1)
tab_results_b$comb2 <- gsub("\\)", "", tab_results_b$comb2)


# Apply the function to both columns
tab_results_b = tab_results_b %>%
 mutate(
  comb1_split = map(comb1, split_combination),
  comb2_split = map(comb2, split_combination),

  fungi1 = map_chr(comb1_split, "fungi"),
  plant1 = map_chr(comb1_split, "plant"),
  fungi2 = map_chr(comb2_split, "fungi"),
  plant2 = map_chr(comb2_split, "plant")
 ) %>%
 select(-comb1_split, -comb2_split)  # Remove the intermediate columns

tab_results_b = tab_results_b %>%
 select(-comb1, -comb2)
```

```
#keep rows plant1=plant2

filtered_results_blwg <- tab_results_b[tab_results_b$plant1 == tab_results_b$plant2, ]


write.csv2(filtered_results_blwg, "C:/Users/Dpao/Desktop/Master's Biology/Master Thesis/#2 semester project/Ericoid Project/MGR_blwg_pairs_3rdmar.csv", row.names=FALSE)


#######################################################################
######################### TOTAL BIOMASS ###############################
#######################################################################

#1. model

#2. emmeans with plant*fungi interaction and only plant

#3. plot MGR

#4. pairwise comparison

######################### model #############################

n = min(dat1$MGR)

h = n*-1+0.01


bm5 = lmer(log(MGR+h) ~fungal_sp * plant_sp + Height.t0 + (1|block), data=dat1)

summary(bm5)

anova(bm5)

plot(bm5)


#save model output

# Extract fixed effects coefficients and statistics

total_model_output <- as.data.frame(summary(bm5)$coefficients)


# Write to CSV

write.csv2(total_model_output, "C:/Users/Dpao/Desktop/Master's Biology/Master Thesis/#2 semester project/Ericoid Project/MGR_total_model_output_5thfeb.csv", row.names=TRUE)


################################# emmeans ###################################

means_total = emmeans::emmeans(bm5, ~fungal_sp*plant_sp, type="response")


means_total

summary(means_total)
```

```
#Add p-value and statistical tests

means_total = update(means_total, infer = c(TRUE, TRUE), null = log(35), type="response",

        calc = c(n = ".wgt."))

summary(means_total)


meas_total = summary(means_total)

meas_total$response


# Write to CSV

write.csv2(meas_total, "C:/Users/Dpao/Desktop/Master's Biology/Master Thesis/#2 semester project/Ericoid Project/MGR_total_emmeans_output_5thfeb.csv", row.names=TRUE)


## emmeans for plant only

means_total_p = emmeans::emmeans(bm5, ~plant_sp, type="response")


means_total_p

summary(means_total_p)


#Add p-value and statistical tests

means_total_p = update(means_total_p, infer = c(TRUE, TRUE), null = log(35), type="response",

        calc = c(n = ".wgt."))

summary(means_total_p)


meas_total_p = summary(means_total_p)


# Write to CSV

write.csv2(meas_total_p, "C:/Users/Dpao/Desktop/Master's Biology/Master Thesis/#2 semester project/Ericoid Project/MGR_total_emmeans_plants_output_5thfeb.csv", row.names=TRUE)


## emmeans for fungi only

means_total_f = emmeans::emmeans(bm5, ~fungal_sp, type="response")


means_total_f

summary(means_total_f)
```

```
#Add p-value and statistical tests

means_total_f = update(means_total_f, infer = c(TRUE, TRUE), null = log(35), type="response",

            calc = c(n = ".wgt."))

summary(means_total_f)


meas_total_f = summary(means_total_f)


# Write to CSV

write.csv2(meas_total_f, "C:/Users/Dpao/Desktop/Master's Biology/Master Thesis/#2 semester project/Ericoid Project/MGR_total_emmeans_fungi_output_5thfeb.csv", row.names=TRUE)


################################ Plot TOTAL BIOMASS ####################################

#add column to specify if error bars are going to be dashed or not

meas_total$lines=ifelse(sign(meas_total$lower.CL)==sign(meas_total$upper.CL),"1","2")


meas_total$plant_sp <- factor(meas_total$plant_sp,levels = c("G. shallon", "V. myrtillus", "P. japonica",

                              "V. vitis-idaea", "C. vulgaris", "V. angustifolium",

                              "K. latifolia", "R. arboreum", "R. ferrugineum"))


#Add Sterile Treatment to Dataset – same as aboveground biomass

# add "Sterile" to fungal species factor levels

meas_total$fungal_sp <- factor(meas_total$fungal_sp,

              levels = c(levels(meas_total$fungal_sp), "Non-inoculated"))


# Create a data frame with all plant species and the Sterile treatment

sterile_rows <- data.frame(

  plant_sp = levels(meas_total$plant_sp),

  fungal_sp = rep("Non-inoculated", length(levels(meas_total$plant_sp)))

)


# Add any other necessary columns with NA or appropriate default values

# Assuming other columns exist in meas_total, add them to sterile_rows with NA

other_cols <- setdiff(names(meas_total), c("plant_sp", "fungal_sp"))

for(col in other_cols) {

  sterile_rows[[col]] <- 0

}
```

```
# Combine the original data with the new sterile rows
meas_total_with_sterile <- rbind(meas_total, sterile_rows)


meas_total_with_sterile$fungal_sp <- factor(meas_total_with_sterile$fungal_sp,
                    levels = c("Non-inoculated","H. gryndleri", "H. hepaticicola_PK 135-3", "H. hepaticicola_UAMH7357/ICMP18553",
                          "K. argillacea", "H. bicolor", "H. variabilis",
                          "O. maius", "Serendipitaceae_sp"))


png("figures/total_MGR/MGR_total_emmeans_updatedtryout.jpg", width = 18, height = 5.4, units ='in', res = 300)
ggplot(data=meas_total_with_sterile, mapping=aes(x=fungal_sp, color=fungal_sp)) +
 facet_grid(cols = vars(plant_sp)) +
 # Split geom_point into two layers - one for Sterile and one for other treatments
 geom_point(data = subset(meas_total_with_sterile, fungal_sp == "Non-inoculated"),
        aes(x= fungal_sp, y=response),
        position=position_dodge(width =1),
        size=1.5,
        alpha=0) +
 geom_point(data = subset(meas_total_with_sterile, fungal_sp != "Non-inoculated"),
        aes(x= fungal_sp, y=response),
        position=position_dodge(width =1),
        size=1.5) +
 geom_errorbar(data = subset(meas_total_with_sterile, fungal_sp == "Non-inoculated"),
          aes(ymin=lower.CL, ymax=upper.CL),
          position=position_dodge(width =1),
          width=.75,
          linewidth=0.7,
          alpha=0) +
 geom_errorbar(data = subset(meas_total_with_sterile, fungal_sp != "Non-inoculated"),
          aes(ymin=lower.CL, ymax=upper.CL, linetype=lines),
          position=position_dodge(width =1),
          width=.75,
          linewidth=0.7) +
 scale_color_manual(values = fungal_colors) +
 theme_classic(base_size = 15) +
```

```
 theme(
   panel.background = element_rect(fill = NA, color = "grey", size = 0.5),
   panel.grid.major = element_blank(),
   panel.grid.minor = element_blank(),
   plot.background = element_rect(fill = "transparent", colour = NA_character_),
   strip.background = element_rect(fill = "grey90", color = "grey", size = 0.5),
   legend.background = element_rect(fill = "transparent"),
   legend.box.background = element_rect(fill = "transparent"),
   legend.key = element_rect(fill = "transparent"),
   axis.title.x = element_blank(),
   axis.text.x = element_blank(),
   axis.ticks.x = element_blank(),
   axis.line = element_line(size = 0.5),
   strip.text = element_text(size = 14),
   plot.title = element_text(hjust = 0.5, size = 16),
   axis.title.y = element_text(size = 16),
   legend.title = element_text(size = 14),
   legend.text = element_text(size = 13)
 ) +
 ylab("Mycorrhizal Growth Response") +
 ylim (-1.5, 25) +
 guides(linetype = "none") +
 labs(color = "Fungal Isolates") +
 geom_hline(yintercept=0, linetype='dotted', col = "black")
dev.off()

############################## pairwise comparisons ###########################
tab_results_t = pairs(means_total)
tab_results_t = as.data.frame(tab_results_t)

tab_results_t = separate(tab_results_t, col=contrast, into=c('comb1', 'comb2'), sep=' - ')
tab_results_t$p_value_stars <- makeStars(tab_results_t$p.value)

#remove "(" and ")" from some of the cells
tab_results_t$comb1 <- gsub("\\(", "", tab_results_t$comb1)
```

```
tab_results_t$comb2 <- gsub("\\(", "", tab_results_t$comb2)

tab_results_t$comb1 <- gsub("\\)", "", tab_results_t$comb1)

tab_results_t$comb2 <- gsub("\\)", "", tab_results_t$comb2)


# Apply the function to both columns

tab_results_t = tab_results_t %>%

  mutate(

    comb1_split = map(comb1, split_combination),

    comb2_split = map(comb2, split_combination),


    fungi1 = map_chr(comb1_split, "fungi"),

    plant1 = map_chr(comb1_split, "plant"),

    fungi2 = map_chr(comb2_split, "fungi"),

    plant2 = map_chr(comb2_split, "plant")

  ) %>%

  select(-comb1_split, -comb2_split)  # Remove the intermediate columns


tab_results_t = tab_results_t %>%

  select(-comb1, -comb2)


# Filter to keep only rows where plant species match

filtered_results <- tab_results_t[tab_results_t$plant1 == tab_results_t$plant2, ]


write.csv2(filtered_results, "C:/Users/Dpao/Desktop/Master's Biology/Master Thesis/#2 semester project/Ericoid Project/MGR_total_pairs_3rdmar.csv", row.names=FALSE)


#################################################################################

########################### RATIO ABVG/BLWG ###################################

a = min(dat1$MGR_ratio)

b = a*-1+0.01


bm6 = lmer(log(MGR_ratio+b)~fungal_sp * plant_sp + Height.t0 + (1|block), data=dat1)

summary(bm6)

ratio_anova = anova(bm6) #interaction is not significant, so plot much simpler and not go to emmeans

plot(bm6)
```

```
#save model output

# Extract fixed effects coefficients and statistics

ratio_model_output <- as.data.frame(summary(bm6)$coefficients)


# Write to CSV

write.csv2(ratio_model_output, "C:/Users/Dpao/Desktop/Master's Biology/Master Thesis/#2 semester project/Ericoid Project/root_shoot_ratio_model_output_5thfeb.csv", row.names=TRUE)

write.csv2(ratio_anova, "C:/Users/Dpao/Desktop/Master's Biology/Master Thesis/#2 semester project/Ericoid Project/root_shoot_ratio_model_anova_5thfeb.csv", row.names=TRUE)


#for fungi

means_ratio_fungi = emmeans::emmeans(bm6, ~fungal_sp, type="response")

means_ratio_fungi

summary(means_ratio_fungi)

#Add p-value and statistical tests

means_ratio_fungi = update(means_ratio_fungi, infer = c(TRUE, TRUE), null = log(35), type="response",

        calc = c(n = ".wgt."))

summary(means_ratio_fungi)

meas_ratio_fungi = summary(means_ratio_fungi)

meas_ratio_fungi$response


# Write to CSV for fungi

write.csv2(meas_ratio_fungi, "C:/Users/Dpao/Desktop/Master's Biology/Master Thesis/#2 semester project/Ericoid Project/root_shoot_ratio_emmeans_fungi_output_5thfeb.csv", row.names=TRUE)


#for plant

means_ratio_plant = emmeans::emmeans(bm6, ~plant_sp, type="response")

means_ratio_plant

summary(means_ratio_plant)


#Add p-value and statistical tests

means_ratio_plant = update(means_ratio_plant, infer = c(TRUE, TRUE), null = log(35), type="response",

          calc = c(n = ".wgt."))

summary(means_ratio_plant)


meas_ratio_plant = summary(means_ratio_plant)
```

```
meas_ratio_plant$response


# Write to CSV for fungi

write.csv2(meas_ratio_plant, "C:/Users/Dpao/Desktop/Master's Biology/Master Thesis/#2 semester project/Ericoid Project/root_shoot_ratio_emmeans_plant_output_5thfeb.csv", row.names=TRUE)


###################### Plots ratio fungi ########################


#add column to specify if error bars are going to be dashed or not

meas_ratio_fungi$lines=ifelse(sign(meas_ratio_fungi$lower.CL)==sign(meas_ratio_fungi$upper.CL),"1","2")




meas_ratio_fungi$fungal_sp <- factor(meas_ratio_fungi$fungal_sp,levels = c("Sterile","H. gryndleri", "H. hepaticicola_PK 135-3", "H. hepaticicola_UAMH7357/ICMP18553",

                                                  "K. argillacea", "H. bicolor", "H. variabilis",

                                                  "O. maius", "Serendipitaceae_sp"))


# code to make plots with ore than one plant species to compare it

ratio_fungi = ggplot(data=meas_ratio_fungi, mapping=aes(x= fungal_sp, color=fungal_sp)) +

 geom_point(aes(x= fungal_sp, y=response), position=position_dodge(width =1),size=1.5, alpha=0.3) +

 geom_errorbar(aes(ymin=lower.CL, ymax=upper.CL, linetype=lines), position=position_dodge(width =1), width=0.4, linewidth=0.7) +

 theme_classic(base_size = 16) +

 theme(panel.grid.major = element_blank(),

     panel.grid.minor = element_blank(),

     plot.background = element_rect(fill = "transparent",

                     colour = NA_character_),

     legend.background = element_rect(fill = "transparent"),

     legend.box.background = element_rect(fill = "transparent"),

     legend.key = element_rect(fill = "transparent"),

     axis.title.x=element_blank(),

     axis.text.x=element_blank(),

     axis.ticks.x=element_blank()) +

 scale_color_manual(values = fungal_colors) +

 theme(panel.background = element_rect(fill = NA, color = "grey"))+

 theme(plot.title = element_text(hjust = 0.5))+
```

```
 ylab("Difference in shoot:root\nratio due to inoculation") +
 guides(linetype = "none") +
 labs(color = "Fungal Isolates") +
 geom_hline(yintercept=0, linetype='dotted', col = "black")

###################### Plots ratio plant ########################
#add column to specify if error bars are going to be dashed or not
meas_ratio_plant$lines=ifelse(sign(meas_ratio_plant$lower.CL)==sign(meas_ratio_plant$upper.CL),"1","2")

meas_ratio_plant$plant_sp <- factor(meas_ratio_plant$plant_sp,levels = c("G. shallon", "V. myrtillus", "P. japonica",
                                               "V. vitis-idaea", "C. vulgaris", "V. angustifolium",
                                               "K. latifolia", "R. arboreum", "R. ferrugineum"))

# code to make plots with more than one plant species to compare it
ratio_plants = ggplot(data=meas_ratio_plant, mapping=aes(x= plant_sp, color=plant_sp)) +
 geom_point(aes(x= plant_sp, y=response), position=position_dodge(width =1),size=1.5, alpha=0.3) +
 geom_errorbar(aes(ymin=lower.CL, ymax=upper.CL, linetype=lines), position=position_dodge(width =1), width=.4, linewidth=0.7) +
 theme_classic(base_size = 16) +
 theme(panel.grid.major = element_blank(),
    panel.grid.minor = element_blank(),
    plot.background = element_rect(fill = "transparent",
                    colour = NA_character_),
    legend.background = element_rect(fill = "transparent"),
    legend.box.background = element_rect(fill = "transparent"),
    legend.key = element_rect(fill = "transparent"),
    axis.title.x=element_blank(),
    axis.text.x=element_blank(),
    axis.ticks.x=element_blank()) +
 theme(panel.background = element_rect(fill = NA, color = "grey"))+
 theme(plot.title = element_text(hjust = 0.5))+
 ylab("Difference in shoot:root\nratio due to inoculation") +
 guides(linetype = "none") +
 labs(color = "Plant species") +
 geom_hline(yintercept=0, linetype='dotted', col = "black")
```

```
plot_ratio = ggarrange(ratio_plants, ratio_fungi, nrow = 1)
png("figures/ratio_both_emmeans.jpg", width = 12, height = 4.5, units ='in', res = 300)
plot_ratio
dev.off()
```

# 5. response_specificity_plots.R

```
library(tidyverse)

################## RESPONSE SPECIFICITY FUNGAL SPECIES ########
#plots for response specificity fungi
create_stacked_barplot <- function(df) {
 # order of species based on positive appearances
 species_order <- df %>%
  arrange(desc(MGR_positive)) %>%
  pull(fungal_sp)

 # Reshape data from wide to long format
 df_long <- df %>%
  mutate(fungal_sp = factor(fungal_sp, levels = species_order)) %>%
  pivot_longer(
   cols = starts_with("MGR_"),
   names_to = "appearance_type",
   values_to = "count"
  ) %>%
  mutate(
   appearance_type = str_remove(appearance_type, "MGR_")
  )

 # Create the plot
 ggplot(df_long, aes(x = fungal_sp, y = count, fill = appearance_type)) +
  geom_bar(stat = "identity", position = "stack") +
  scale_fill_manual(
   values = c(
```

```
      "positive" = "#2ECC71",
      "negative" = "#E74C3C",
      "ns" = "#95A5A6"
    ),
    name = "",
    labels = c(
      "positive" = "MGR > 0",
      "negative" = "MGR < 0",
      "ns" = "MGR Non-significant"
    )
  ) +
  scale_y_continuous(breaks = function(x) seq(from = 0, to = ceiling(max(x)), by = 1)) +  # Integer breaks
  theme_classic(base_size = 16) +  # Increase base font size
  labs(
    title = "Fungal isolate response specificity",
    x = "",
    y = "Number of plant species"
  ) +
  theme(
    axis.text.x = element_text(angle = 70, hjust = 1, size = 12),
    axis.text.y = element_text(size = 12),
    axis.title = element_text(size = 13),
    legend.text = element_text(size = 13),
    legend.title = element_text(size = 1),
    title = element_text(size = 13),
    panel.grid.major.x = element_blank(),
    panel.border = element_blank(),
    legend.position = "right"
  )
}

##### LOAD DATA
### total MGR
dat_total_fungi = read.csv2("response_specificity_total_fungi.csv")
#str(dat_total_fungi)
```

```
#dat_total_fungi


### abvg MGR
dat_abvg_fungi = read.csv2("response_specificity_abvg_fungi.csv")
#str(dat_abvg_fungi)
#dat_abvg_fungi


### blwg MGR
dat_blwg_fungi = read.csv2("response_specificity_blwg_fungi.csv")
#str(dat_blwg_fungi)
#dat_blwg_fungi


### CREATE PLOTS
## total MGR
plot_total_fungi <- create_stacked_barplot(dat_total_fungi)
#print(plot_total_fungi)


png("figures/response_specificity/response_specificity_total_fungi.jpg", width = 7, height = 7, units ='in', res = 300)
plot_total_fungi
dev.off()


## abvg MGR
plot_abvg_fungi <- create_stacked_barplot(dat_abvg_fungi)
#print(plot_abvg_fungi)


png("figures/response_specificity/response_specificity_abvg_fungi.jpg", width = 7, height = 7, units ='in', res = 300)
plot_abvg_fungi
dev.off()


## blwg MGR
plot_blwg_fungi <- create_stacked_barplot(dat_blwg_fungi)
#print(plot_blwg_fungi)


png("figures/response_specificity/response_specificity_blwg_fungi.jpg", width = 7, height = 7, units ='in', res = 300)
plot_blwg_fungi
```

```
dev.off()


###############################################################################
#################### RESPONSE SPECIFICITY FUNGAL SPECIES #####################
###############################################################################
#plots for response specificity plant
create_stacked_barplot <- function(df) {
 # order of species based on positive appearances
 species_order <- df %>%
  arrange(desc(MGR_positive)) %>%
  pull(plant_sp)

 # Reshape data from wide to long format
 df_long <- df %>%
  mutate(plant_sp = factor(plant_sp, levels = species_order)) %>%
  pivot_longer(
   cols = starts_with("MGR_"),
   names_to = "appearance_type",
   values_to = "count"
  ) %>%
  mutate(
   appearance_type = str_remove(appearance_type, "MGR_")
  )

 # Create the plot
 ggplot(df_long, aes(x = plant_sp, y = count, fill = appearance_type)) +
  geom_bar(stat = "identity", position = "stack") +
  scale_fill_manual(
   values = c(
    "positive" = "#2ECC71",
    "negative" = "#E74C3C",
    "ns" = "#95A5A6"
   ),
   name = "",
   labels = c(
```

```
    "positive" = "MGR > 0",
    "negative" = "MGR < 0",
    "ns" = "MGR Non-significant"
   )
  ) +
  scale_y_continuous(breaks = function(x) seq(from = 0, to = ceiling(max(x)), by = 1)) +  # Integer breaks
  theme_classic(base_size = 16) +  # Increase base font size
  labs(
   title = "Plant species response specificity",
   x = "",
   y = "Number of fungal species"
  ) +
  theme(
   axis.text.x = element_text(angle = 70, hjust = 1, size = 12),
   axis.text.y = element_text(size = 12),
   axis.title = element_text(size = 13),
   title = element_text(size = 13),
   panel.grid.major.x = element_blank(),
   panel.border = element_blank(),
   legend.position = "none"
  )
}

##### LOAD DATA
### total MGR
dat_total_plant = read.csv2("response_specificity_total_plant.csv")
#str(dat_total_plant)
#dat_total_plant

### abvg MGR
dat_abvg_plant = read.csv2("response_specificity_abvg_plant.csv")
#str(dat_abvg_plant)
#dat_abvg_plant

### blwg MGR
```

```
dat_blwg_plant = read.csv2("response_specificity_blwg_plant.csv")
#str(dat_blwg_plant)
#dat_blwg_plant

### CREATE PLOTS
## total MGR
plot_total_plant <- create_stacked_barplot(dat_total_plant)
#print(plot_total_plant)

png("figures/response_specificity/response_specificity_total_plant.jpg", width = 7, height = 7, units ='in', res = 300)
plot_total_plant
dev.off()

## abvg MGR
plot_abvg_plant <- create_stacked_barplot(dat_abvg_plant)
#print(plot_abvg_plant)

png("figures/response_specificity/response_specificity_abvg_plant.jpg", width = 7, height = 7, units ='in', res = 300)
plot_abvg_plant
dev.off()

## blwg MGR
plot_blwg_plant <- create_stacked_barplot(dat_blwg_plant)
#print(plot_blwg_plant)

png("figures/response_specificity/response_specificity_blwg_plant.jpg", width = 7, height = 7, units ='in', res = 300)
plot_blwg_plant
dev.off()


library(egg)
plot_total = ggarrange(plot_total_plant, plot_total_fungi, nrow = 1)
png("figures/response_specificity/response_specificity_total.jpg", width = 10, height = 6, units ='in', res = 300)
plot_total
dev.off()
```

```
#abvg
plot_abvg = ggarrange(plot_abvg_plant, plot_abvg_fungi, nrow = 1)
png("figures/response_specificity/response_specificity_abvg.jpg", width = 10, height = 6, units ='in', res = 300)
plot_abvg
dev.off()

#blwg
plot_blwg = ggarrange(plot_blwg_plant, plot_blwg_fungi, nrow = 1)
png("figures/response_specificity/response_specificity_blwg.jpg", width = 10, height = 6, units ='in', res = 300)
plot_blwg
dev.off()
```

## 6. ericoid_phylogeny.R

```
library(ape)
packageVersion("ape")
library(tidyverse)
packageVersion("tidyverse")
library(abdiv)
packageVersion("abdiv")
library(doParallel)
packageVersion("doParallel")
library(foreach)
packageVersion("foreach")
library(pez)
packageVersion("pez")
library(phylosignal)
library(phytools)
library(phylobase)
library(reshape2)
library(xts)


####################################
```

```
############# make dat ##############
#####################################

plants = c("Vaccinium_angustifolium", "Calluna_vulgaris",
        "Rhododendron_ferrugineum", "Rhododendron_arboreum",
        "Vaccinium_myrtillus", "Kalmia_latifolia", "Pieris_japonica",
        "Vaccinium_vitis-idaea", "Gaultheria_shallon")
plant_sp = as.data.frame(plants)

#####################################
######## Build tree per plot ########
#####################################

load("GBOTB.extended.rda")
GBOTB.extended.congen <- congeneric.merge(GBOTB.extended,plants, split = "_", cite = FALSE)
alltreesp <- c(GBOTB.extended.congen$tip.label) %>% as.data.frame()
colnames(alltreesp) <- "plants"

registerDoParallel(48)
filename = paste("trees/tree_", "plants_ericoid", sep = "")
nodrop <- plant_sp%>% select(plants)
todrop <- setdiff(alltreesp, nodrop) %>% as.vector()
todrop <- c(t(todrop))
p.tree <- drop.tip(GBOTB.extended.congen, unlist(todrop))
filepath = paste(filename, ".tre", sep = "")
write.tree(p.tree, filepath)

##############################

p.tree = read.tree("trees/tree_plants_ericoid.tre")
plot.phylo(p.tree)

##############################################################
###################### ABOVEGROUND ##########################
##############################################################
```

```
dat_abvg = read.csv2("MGR_response_abvg.csv")
str(dat_abvg)

dat_abvg = dat_abvg%>%
  select(-c("df", "n","lower.CL", "upper.CL", "null", "t.ratio", "p.value", "lines"))
str(dat_abvg)
summary(dat_abvg)

dat_abvg <- transform(dat_abvg,plant_sp=gsub(pattern="C_vulgaris", replacement="Calluna_vulgaris", plant_sp))
dat_abvg <- transform(dat_abvg,plant_sp=gsub(pattern="G_shallon", replacement="Gaultheria_shallon", plant_sp))
dat_abvg <- transform(dat_abvg,plant_sp=gsub(pattern="K_latifolia", replacement="Kalmia_latifolia", plant_sp))
dat_abvg <- transform(dat_abvg,plant_sp=gsub(pattern="P_japonica", replacement="Pieris_japonica", plant_sp))
dat_abvg <- transform(dat_abvg,plant_sp=gsub(pattern="R_arboreum", replacement="Rhododendron_arboreum", plant_sp))
dat_abvg <- transform(dat_abvg,plant_sp=gsub(pattern="R_ferrugineum", replacement="Rhododendron_ferrugineum", plant_sp))
dat_abvg <- transform(dat_abvg,plant_sp=gsub(pattern="V_angustifolium", replacement="Vaccinium_angustifolium", plant_sp))
dat_abvg <- transform(dat_abvg,plant_sp=gsub(pattern="V_myrtillus", replacement="Vaccinium_myrtillus", plant_sp))
dat_abvg <- transform(dat_abvg,plant_sp=gsub(pattern="V_vitis-idaea", replacement="Vaccinium_vitis-idaea", plant_sp))

##### CREATE HEAT MAP OF MGR
dat_abvg1 = dat_abvg%>% select(-c("SE"))
response_abvg <- dcast(data = dat_abvg1, formula = plant_sp ~ fungal_sp)
rownames(response_abvg) <- response_abvg$plant_sp
response_abvg = response_abvg%>% select(-c("plant_sp"))

#min(response_abvg)
#max(response_abvg)

par(mar=c(0,0,0,0))

png("figures/heatmap_abvg.jpg", width = 10, height = 10, units ='in', res = 300)
phylo.heatmap(p.tree,response_abvg,
        split=c(0.8,0.7),fsize=c(1.5,0.5,1),
        standardize=FALSE,pts=FALSE, legend=TRUE, labels=TRUE)
dev.off()
```

```
par(mar=c(5.1,4.1,4.1,2.1)) ## reset margins to default

###### PHYLOGENETIC SIGNAL FOR EACH FUNGAL SPECIES
############## H. bicolor
dat_bicolor_abvg = dat_abvg %>% subset(dat_abvg$fungal_sp=="H_bicolor") %>%
  remove_rownames %>% column_to_rownames(var="plant_sp")

response_bicolor_abvg = setNames(dat_bicolor_abvg$response, rownames(dat_bicolor_abvg))
se_bicolor_abvg = setNames(dat_bicolor_abvg$SE, rownames(dat_bicolor_abvg))

# K-test
set.seed(123)
K_bicolor_abvg <-phylosig(p.tree, response_bicolor_abvg, se=se_bicolor_abvg,method="K", test=TRUE)
#print(K_bicolor_abvg); plot(K_bicolor_abvg)
# Pagel's Lambda
set.seed(123)
lambda_bicolor_abvg <-phylosig(p.tree, response_bicolor_abvg, se=se_bicolor_abvg,method="lambda", test=TRUE)
#print(lambda_bicolor_abvg); plot(lambda_bicolor_abvg)

############## H_gryndleri
dat_gryndleri_abvg = dat_abvg %>% subset(dat_abvg$fungal_sp=="H_gryndleri") %>%
  remove_rownames %>% column_to_rownames(var="plant_sp")

response_gryndleri_abvg = setNames(dat_gryndleri_abvg$response, rownames(dat_gryndleri_abvg))
se_gryndleri_abvg = setNames(dat_gryndleri_abvg$SE, rownames(dat_gryndleri_abvg))

# K-test
set.seed(123)
K_gryndleri_abvg <-phylosig(p.tree, response_gryndleri_abvg, se=se_gryndleri_abvg, method="K", test=TRUE)
#print(K_gryndleri_abvg); plot(K_gryndleri_abvg)
# Pagel's Lambda
set.seed(123)
```

```
lambda_gryndleri_abvg <-phylosig(p.tree, response_gryndleri_abvg, se=se_gryndleri_abvg, method="lambda", test=TRUE)
#print(lambda_gryndleri_abvg); plot(lambda_gryndleri_abvg)


############## H_hepaticicola_1
dat_hep1_abvg = dat_abvg %>% subset(dat_abvg$fungal_sp=="H_hepaticicola_1") %>%
  remove_rownames %>% column_to_rownames(var="plant_sp")

response_hep1_abvg = setNames(dat_hep1_abvg$response, rownames(dat_hep1_abvg))
se_hep1_abvg = setNames(dat_hep1_abvg$SE, rownames(dat_hep1_abvg))

# K-test
set.seed(123)
K_hep1_abvg <-phylosig(p.tree, response_hep1_abvg, se=se_hep1_abvg, method="K", test=TRUE)
#print(K_hep1_abvg); plot(K_hep1_abvg)
# Pagel's Lambda
set.seed(123)
lambda_hep1_abvg <-phylosig(p.tree, response_hep1_abvg, se=se_hep1_abvg, method="lambda", test=TRUE)
#print(lambda_hep1_abvg); plot(lambda_hep1_abvg)


############## H_hepaticicola_2
dat_hep2_abvg = dat_abvg %>% subset(dat_abvg$fungal_sp=="H_hepaticicola_2") %>%
  remove_rownames %>% column_to_rownames(var="plant_sp")

response_hep2_abvg = setNames(dat_hep2_abvg$response, rownames(dat_hep2_abvg))
se_hep2_abvg = setNames(dat_hep2_abvg$SE, rownames(dat_hep2_abvg))

# K-test
set.seed(123)
K_hep2_abvg <-phylosig(p.tree, response_hep2_abvg, se=se_hep2_abvg,method="K", test=TRUE)
#print(K_hep2_abvg); plot(K_hep2_abvg)
# Pagel's Lambda
set.seed(123)
lambda_hep2_abvg <-phylosig(p.tree, response_hep2_abvg, se=se_hep2_abvg, method="lambda", test=TRUE)
```

```
#print(lambda_hep2_abvg); plot(lambda_hep2_abvg)



############## H_variabilis
dat_variabilis_abvg = dat_abvg %>% subset(dat_abvg$fungal_sp=="H_variabilis") %>%
  remove_rownames %>% column_to_rownames(var="plant_sp")

response_variabilis_abvg = setNames(dat_variabilis_abvg$response, rownames(dat_variabilis_abvg))
se_variabilis_abvg = setNames(dat_variabilis_abvg$SE, rownames(dat_variabilis_abvg))

# K-test
set.seed(123)
K_variabilis_abvg <-phylosig(p.tree, response_variabilis_abvg, se=se_variabilis_abvg, method="K", test=TRUE)
#print(K_variabilis_abvg); plot(K_variabilis_abvg)
# Pagel's Lambda
set.seed(123)
lambda_variabilis_abvg <-phylosig(p.tree, response_variabilis_abvg, se=se_variabilis_abvg, method="lambda", test=TRUE)
#print(lambda_variabilis_abvg); plot(lambda_variabilis_abvg)



############## K_argillacea
dat_argillacea_abvg = dat_abvg %>% subset(dat_abvg$fungal_sp=="K_argillacea") %>%
  remove_rownames %>% column_to_rownames(var="plant_sp")

response_argillacea_abvg = setNames(dat_argillacea_abvg$response, rownames(dat_argillacea_abvg))
se_argillacea_abvg = setNames(dat_argillacea_abvg$SE, rownames(dat_argillacea_abvg))

# K-test
set.seed(123)
K_argillacea_abvg <-phylosig(p.tree, response_argillacea_abvg, se=se_argillacea_abvg, method="K", test=TRUE)
#print(K_argillacea_abvg); plot(K_argillacea_abvg)
# Pagel's Lambda
set.seed(123)
lambda_argillacea_abvg <-phylosig(p.tree, response_argillacea_abvg, se=se_argillacea_abvg,method="lambda", test=TRUE)
#print(lambda_argillacea_abvg); plot(lambda_argillacea_abvg)
```

```
############## O_maius

dat_maius_abvg = dat_abvg %>% subset(dat_abvg$fungal_sp=="O_maius") %>%
  remove_rownames %>% column_to_rownames(var="plant_sp")

response_maius_abvg = setNames(dat_maius_abvg$response, rownames(dat_maius_abvg))
se_maius_abvg = setNames(dat_maius_abvg$SE, rownames(dat_maius_abvg))

# K-test
set.seed(123)
K_maius_abvg <-phylosig(p.tree, response_maius_abvg, se=se_maius_abvg, method="K", test=TRUE)
#print(K_maius_abvg); plot(K_maius_abvg)
# Pagel's Lambda
set.seed(123)
lambda_maius_abvg <-phylosig(p.tree, response_maius_abvg, se=se_maius_abvg, method="lambda", test=TRUE)
#print(lambda_maius_abvg); plot(lambda_maius_abvg)



############## Serendipitaceae_sp
dat_Serendipitaceae_abvg = dat_abvg %>% subset(dat_abvg$fungal_sp=="Serendipitaceae_sp") %>%
  remove_rownames %>% column_to_rownames(var="plant_sp")

response_Serendipitaceae_abvg = setNames(dat_Serendipitaceae_abvg$response, rownames(dat_Serendipitaceae_abvg))
se_Serendipitaceae_abvg = setNames(dat_Serendipitaceae_abvg$SE, rownames(dat_Serendipitaceae_abvg))

# K-test
set.seed(123)
K_Serendipitaceae_abvg <-phylosig(p.tree, response_Serendipitaceae_abvg, se=se_Serendipitaceae_abvg, method="K", test=TRUE)
#print(K_Serendipitaceae_abvg); plot(K_Serendipitaceae_abvg)
# Pagel's Lambda
set.seed(123)
lambda_Serendipitaceae_abvg <-phylosig(p.tree, response_Serendipitaceae_abvg, se=se_Serendipitaceae_abvg,
method="lambda", test=TRUE)
#print(lambda_Serendipitaceae_abvg); plot(lambda_Serendipitaceae_abvg)
```

```
### DATAFRAME WITH RESULTS FROM K AND LAMBDA TESTS FOR ALL FUNGI

column_names = c("K_test", "K_p_value", "Lambda_test", "Lambda_p_value")

H_bicolor_abvg = c(K_bicolor_abvg$K, K_bicolor_abvg$P, lambda_bicolor_abvg$lambda, lambda_bicolor_abvg$P)

H_gryndleri_abvg = c(K_gryndleri_abvg$K, K_gryndleri_abvg$P, lambda_gryndleri_abvg$lambda, lambda_gryndleri_abvg$P)

H_hepaticicola_1_abvg = c(K_hep1_abvg$K, K_hep1_abvg$P, lambda_hep1_abvg$lambda, lambda_hep1_abvg$P)

H_hepaticicola_2_abvg = c(K_hep2_abvg$K, K_hep2_abvg$P, lambda_hep2_abvg$lambda, lambda_hep2_abvg$P)

H_variabilis_abvg = c(K_variabilis_abvg$K, K_variabilis_abvg$P, lambda_variabilis_abvg$lambda, lambda_variabilis_abvg$P)

K_argillacea_abvg = c(K_argillacea_abvg$K, K_argillacea_abvg$P, lambda_argillacea_abvg$lambda, lambda_argillacea_abvg$P)

O_maius_abvg = c(K_maius_abvg$K, K_maius_abvg$P, lambda_maius_abvg$lambda, lambda_maius_abvg$P)

Serendipitaceae_sp_abvg = c(K_Serendipitaceae_abvg$K, K_Serendipitaceae_abvg$P, lambda_Serendipitaceae_abvg$lambda,
lambda_Serendipitaceae_abvg$P)


df_abvg <- as.data.frame(rbind(H_bicolor_abvg, H_gryndleri_abvg, H_hepaticicola_1_abvg,

             H_hepaticicola_2_abvg, H_variabilis_abvg, K_argillacea_abvg,

             O_maius_abvg, Serendipitaceae_sp_abvg))

colnames(df_abvg)=column_names

df_abvg

write.csv2(df_abvg, "C:/Users/Dpao/Desktop/Master's Biology/Master Thesis/#2 semester project/Ericoid
Project/phylogenetic_analysis_abvg.csv", row.names=TRUE)


###############################################################

###################### BELOWGROUND ##########################

###############################################################


dat_blwg = read.csv2("MGR_response_blwg.csv")

str(dat_blwg)


dat_blwg = dat_blwg%>%

  select(-c("df", "n","lower.CL", "upper.CL", "null", "t.ratio", "p.value", "lines"))

str(dat_blwg)

summary(dat_blwg)


dat_blwg <- transform(dat_blwg,plant_sp=gsub(pattern="C_vulgaris", replacement="Calluna_vulgaris", plant_sp))

dat_blwg <- transform(dat_blwg,plant_sp=gsub(pattern="G_shallon", replacement="Gaultheria_shallon", plant_sp))
```

```
dat_blwg <- transform(dat_blwg,plant_sp=gsub(pattern="K_latifolia", replacement="Kalmia_latifolia", plant_sp))

dat_blwg <- transform(dat_blwg,plant_sp=gsub(pattern="P_japonica", replacement="Pieris_japonica", plant_sp))

dat_blwg <- transform(dat_blwg,plant_sp=gsub(pattern="R_arboreum", replacement="Rhododendron_arboreum", plant_sp))

dat_blwg <- transform(dat_blwg,plant_sp=gsub(pattern="R_ferrugineum", replacement="Rhododendron_ferrugineum", plant_sp))

dat_blwg <- transform(dat_blwg,plant_sp=gsub(pattern="V_angustifolium", replacement="Vaccinium_angustifolium", plant_sp))

dat_blwg <- transform(dat_blwg,plant_sp=gsub(pattern="V_myrtillus", replacement="Vaccinium_myrtillus", plant_sp))

dat_blwg <- transform(dat_blwg,plant_sp=gsub(pattern="V_vitis-idaea", replacement="Vaccinium_vitis-idaea", plant_sp))


##### CREATE HEAT MAP OF MGR


dat_blwg1 = dat_blwg%>% select(-c("SE"))

response_blwg <- dcast(data = dat_blwg1, formula = plant_sp ~ fungal_sp)

rownames(response_blwg) <- response_blwg$plant_sp

response_blwg = response_blwg%>% select(-c("plant_sp"))


#min(response_blwg)

#max(response_blwg)


par(mar=c(0,0,0,0))


png("figures/heatmap_blwg.jpg", width = 10, height = 10, units ='in', res = 300)

phylo.heatmap(p.tree,response_blwg,

        split=c(0.8,0.7),fsize=c(1.5,0.5,1),

        standardize=FALSE,pts=FALSE, legend=TRUE, labels=TRUE)

dev.off()


par(mar=c(5.1,4.1,4.1,2.1)) ## reset margins to default



###### PHYLOGENETIC SIGNAL FOR EACH FUNGAL SPECIES

############## H. bicolor

dat_bicolor_blwg = dat_blwg %>% subset(dat_blwg$fungal_sp=="H_bicolor") %>%

  remove_rownames %>% column_to_rownames(var="plant_sp")


response_bicolor_blwg = setNames(dat_bicolor_blwg$response, rownames(dat_bicolor_blwg))
```

```
se_bicolor_blwg = setNames(dat_bicolor_blwg$SE, rownames(dat_bicolor_blwg))

# K-test
set.seed(123)
K_bicolor_blwg <-phylosig(p.tree, response_bicolor_blwg, se=se_bicolor_blwg, method="K", test=TRUE)
#print(K_bicolor_blwg); plot(K_bicolor_blwg)
# Pagel's Lambda
set.seed(123)
lambda_bicolor_blwg <-phylosig(p.tree, response_bicolor_blwg, se=se_bicolor_blwg, method="lambda", test=TRUE)
#print(lambda_bicolor_blwg); plot(lambda_bicolor_blwg)


############## H_gryndleri
dat_gryndleri_blwg = dat_blwg %>% subset(dat_blwg$fungal_sp=="H_gryndleri") %>%
  remove_rownames %>% column_to_rownames(var="plant_sp")

response_gryndleri_blwg = setNames(dat_gryndleri_blwg$response, rownames(dat_gryndleri_blwg))
se_gryndleri_blwg = setNames(dat_gryndleri_blwg$SE, rownames(dat_gryndleri_blwg))

# K-test
set.seed(123)
K_gryndleri_blwg <-phylosig(p.tree, response_gryndleri_blwg, se=se_gryndleri_blwg, method="K", test=TRUE)
#print(K_gryndleri_blwg); plot(K_gryndleri_blwg)
# Pagel's Lambda
set.seed(123)
lambda_gryndleri_blwg <-phylosig(p.tree, response_gryndleri_blwg, se=se_gryndleri_blwg, method="lambda", test=TRUE)
#print(lambda_gryndleri_blwg); plot(lambda_gryndleri_blwg)


############## H_hepaticicola_1
dat_hep1_blwg = dat_blwg %>% subset(dat_blwg$fungal_sp=="H_hepaticicola_1") %>%
  remove_rownames %>% column_to_rownames(var="plant_sp")

response_hep1_blwg = setNames(dat_hep1_blwg$response, rownames(dat_hep1_blwg))
se_hep1_blwg = setNames(dat_hep1_blwg$SE, rownames(dat_hep1_blwg))
```

```
# K-test
set.seed(123)
K_hep1_blwg <-phylosig(p.tree, response_hep1_blwg, se=se_hep1_blwg, method="K", test=TRUE)
#print(K_hep1_blwg); plot(K_hep1_blwg)
# Pagel's Lambda
set.seed(123)
lambda_hep1_blwg <-phylosig(p.tree, response_hep1_blwg, se=se_hep1_blwg, method="lambda", test=TRUE)
#print(lambda_hep1_blwg); plot(lambda_hep1_blwg)


############## H_hepaticicola_2
dat_hep2_blwg = dat_blwg %>% subset(dat_blwg$fungal_sp=="H_hepaticicola_2") %>%
  remove_rownames %>% column_to_rownames(var="plant_sp")

response_hep2_blwg = setNames(dat_hep2_blwg$response, rownames(dat_hep2_blwg))
se_hep2_blwg = setNames(dat_hep2_blwg$SE, rownames(dat_hep2_blwg))

# K-test
set.seed(123)
K_hep2_blwg <-phylosig(p.tree, response_hep2_blwg, se=se_hep2_blwg, method="K", test=TRUE)
#print(K_hep2_blwg); plot(K_hep2_blwg)
# Pagel's Lambda
set.seed(123)
lambda_hep2_blwg <-phylosig(p.tree, response_hep2_blwg, se=se_hep2_blwg, method="lambda", test=TRUE)
#print(lambda_hep2_blwg); plot(lambda_hep2_blwg)


############## H_variabilis
dat_variabilis_blwg = dat_blwg %>% subset(dat_blwg$fungal_sp=="H_variabilis") %>%
  remove_rownames %>% column_to_rownames(var="plant_sp")

response_variabilis_blwg = setNames(dat_variabilis_blwg$response, rownames(dat_variabilis_blwg))
se_variabilis_blwg = setNames(dat_variabilis_blwg$SE, rownames(dat_variabilis_blwg))
```

```
# K-test
set.seed(123)
K_variabilis_blwg <-phylosig(p.tree, response_variabilis_blwg, se=se_variabilis_blwg, method="K", test=TRUE)
#print(K_variabilis_blwg); plot(K_variabilis_blwg)
# Pagel's Lambda
set.seed(123)
lambda_variabilis_blwg <-phylosig(p.tree, response_variabilis_blwg, se=se_variabilis_blwg, method="lambda", test=TRUE)
#print(lambda_variabilis_blwg); plot(lambda_variabilis_blwg)


############## K_argillacea
dat_argillacea_blwg = dat_blwg %>% subset(dat_blwg$fungal_sp=="K_argillacea") %>%
  remove_rownames %>% column_to_rownames(var="plant_sp")

response_argillacea_blwg = setNames(dat_argillacea_blwg$response, rownames(dat_argillacea_blwg))
se_argillacea_blwg = setNames(dat_argillacea_blwg$SE, rownames(dat_argillacea_blwg))

# K-test
set.seed(123)
K_argillacea_blwg <-phylosig(p.tree, response_argillacea_blwg, se=se_argillacea_blwg, method="K", test=TRUE)
#print(K_argillacea_blwg); plot(K_argillacea_blwg)
# Pagel's Lambda
set.seed(123)
lambda_argillacea_blwg <-phylosig(p.tree, response_argillacea_blwg, se=se_argillacea_blwg, method="lambda", test=TRUE)
#print(lambda_argillacea_blwg); plot(lambda_argillacea_blwg)


############## O_maius
dat_maius_blwg = dat_blwg %>% subset(dat_blwg$fungal_sp=="O_maius") %>%
  remove_rownames %>% column_to_rownames(var="plant_sp")

response_maius_blwg = setNames(dat_maius_blwg$response, rownames(dat_maius_blwg))
se_maius_blwg = setNames(dat_maius_blwg$SE, rownames(dat_maius_blwg))

# K-test
```

```
set.seed(123)

K_maius_blwg <-phylosig(p.tree, response_maius_blwg, se=se_maius_blwg, method="K", test=TRUE)

#print(K_maius_blwg); plot(K_maius_blwg)

# Pagel's Lambda

set.seed(123)

lambda_maius_blwg <-phylosig(p.tree, response_maius_blwg, se=se_maius_blwg, method="lambda", test=TRUE)

#print(lambda_maius_blwg); plot(lambda_maius_blwg)



############## Serendipitaceae_sp

dat_Serendipitaceae_blwg = dat_blwg %>% subset(dat_blwg$fungal_sp=="Serendipitaceae_sp") %>%

  remove_rownames %>% column_to_rownames(var="plant_sp")


response_Serendipitaceae_blwg = setNames(dat_Serendipitaceae_blwg$response, rownames(dat_Serendipitaceae_blwg))

se_Serendipitaceae_blwg = setNames(dat_Serendipitaceae_blwg$SE, rownames(dat_Serendipitaceae_blwg))


# K-test

set.seed(123)

K_Serendipitaceae_blwg <-phylosig(p.tree, response_Serendipitaceae_blwg, se=se_Serendipitaceae_blwg, method="K", test=TRUE)

#print(K_Serendipitaceae_blwg); plot(K_Serendipitaceae_blwg)

# Pagel's Lambda

set.seed(123)

lambda_Serendipitaceae_blwg <-phylosig(p.tree, response_Serendipitaceae_blwg, se=se_Serendipitaceae_blwg, method="lambda", test=TRUE)

#print(lambda_Serendipitaceae_blwg); plot(lambda_Serendipitaceae_blwg)




### DATAFRAME WITH RESULTS FROM K AND LAMBDA TESTS FOR ALL FUNGI

column_names = c("K_test", "K_p_value", "Lambda_test", "Lambda_p_value")

H_bicolor_blwg = c(K_bicolor_blwg$K, K_bicolor_blwg$P, lambda_bicolor_blwg$lambda, lambda_bicolor_blwg$P)

H_gryndleri_blwg = c(K_gryndleri_blwg$K, K_gryndleri_blwg$P, lambda_gryndleri_blwg$lambda, lambda_gryndleri_blwg$P)

H_hepaticicola_1_blwg = c(K_hep1_blwg$K, K_hep1_blwg$P, lambda_hep1_blwg$lambda, lambda_hep1_blwg$P)

H_hepaticicola_2_blwg = c(K_hep2_blwg$K, K_hep2_blwg$P, lambda_hep2_blwg$lambda, lambda_hep2_blwg$P)

H_variabilis_blwg = c(K_variabilis_blwg$K, K_variabilis_blwg$P, lambda_variabilis_blwg$lambda, lambda_variabilis_blwg$P)

K_argillacea_blwg = c(K_argillacea_blwg$K, K_argillacea_blwg$P, lambda_argillacea_blwg$lambda, lambda_argillacea_blwg$P)
```

```
O_maius_blwg = c(K_maius_blwg$K, K_maius_blwg$P, lambda_maius_blwg$lambda, lambda_maius_blwg$P)

Serendipitaceae_sp_blwg = c(K_Serendipitaceae_blwg$K, K_Serendipitaceae_blwg$P, lambda_Serendipitaceae_blwg$lambda, lambda_Serendipitaceae_blwg$P)


df_blwg <- as.data.frame(rbind(H_bicolor_blwg, H_gryndleri_blwg, H_hepaticicola_1_blwg,

                        H_hepaticicola_2_blwg, H_variabilis_blwg, K_argillacea_blwg,

                        O_maius_blwg, Serendipitaceae_sp_blwg))

colnames(df_blwg)=column_names

df_blwg

write.csv2(df_blwg, "C:/Users/Dpao/Desktop/Master's Biology/Master Thesis/#2 semester project/Ericoid Project/phylogenetic_analysis_blwg.csv", row.names=TRUE)


#################################################################

##################### TOTAL BIOMASS #########################

#################################################################

dat_t = read.csv2("MGR_response_total.csv")

str(dat_t)


dat_t = dat_t%>%

  select(-c("df", "n","lower.CL", "upper.CL", "null", "t.ratio", "p.value", "lines"))

str(dat_t)

summary(dat_t)


dat_t <- transform(dat_t,plant_sp=gsub(pattern="C_vulgaris", replacement="Calluna_vulgaris", plant_sp))

dat_t <- transform(dat_t,plant_sp=gsub(pattern="G_shallon", replacement="Gaultheria_shallon", plant_sp))

dat_t <- transform(dat_t,plant_sp=gsub(pattern="K_latifolia", replacement="Kalmia_latifolia", plant_sp))

dat_t <- transform(dat_t,plant_sp=gsub(pattern="P_japonica", replacement="Pieris_japonica", plant_sp))

dat_t <- transform(dat_t,plant_sp=gsub(pattern="R_arboreum", replacement="Rhododendron_arboreum", plant_sp))

dat_t <- transform(dat_t,plant_sp=gsub(pattern="R_ferrugineum", replacement="Rhododendron_ferrugineum", plant_sp))

dat_t <- transform(dat_t,plant_sp=gsub(pattern="V_angustifolium", replacement="Vaccinium_angustifolium", plant_sp))

dat_t <- transform(dat_t,plant_sp=gsub(pattern="V_myrtillus", replacement="Vaccinium_myrtillus", plant_sp))

dat_t <- transform(dat_t,plant_sp=gsub(pattern="V_vitis-idaea", replacement="Vaccinium_vitis-idaea", plant_sp))


##### CREATE HEAT MAP OF MGR

dat_t1 = dat_t%>% select(-c("SE"))

response_t <- dcast(data = dat_t1, formula = plant_sp ~ fungal_sp)
```

```
rownames(response_t) <- response_t$plant_sp
response_t = response_t%>% select(-c("plant_sp"))

#min(response_t)
#max(response_t)

par(mar=c(0,0,0,0))

png("figures/heatmap_total.jpg", width = 10, height = 10, units ='in', res = 300)
phylo.heatmap(p.tree,response_t,
        split=c(0.8,0.7),fsize=c(1.5,0.5,1),
        standardize=FALSE,pts=FALSE, legend=TRUE, labels=TRUE)
dev.off()

par(mar=c(5.1,4.1,4.1,2.1)) ## reset margins to default



###### PHYLOGENETIC SIGNAL FOR EACH FUNGAL SPECIES
############## H. bicolor
dat_bicolor_t = dat_t %>% subset(dat_t$fungal_sp=="H_bicolor") %>%
  remove_rownames %>% column_to_rownames(var="plant_sp")

response_bicolor_t = setNames(dat_bicolor_t$response, rownames(dat_bicolor_t))
se_bicolor_t = setNames(dat_bicolor_t$SE, rownames(dat_bicolor_t))

# K-test
set.seed(123)
K_bicolor_t <-phylosig(p.tree, response_bicolor_t, se=se_bicolor_t, method="K", test=TRUE)
#print(K_bicolor_t); plot(K_bicolor_t)
# Pagel's Lambda
set.seed(123)
lambda_bicolor_t <-phylosig(p.tree, response_bicolor_t, se=se_bicolor_t, method="lambda", test=TRUE)
#print(lambda_bicolor_t); plot(lambda_bicolor_t)
```

```
############## H_gryndleri
dat_gryndleri_t = dat_t %>% subset(dat_t$fungal_sp=="H_gryndleri") %>%
  remove_rownames %>% column_to_rownames(var="plant_sp")

response_gryndleri_t = setNames(dat_gryndleri_t$response, rownames(dat_gryndleri_t))
se_gryndleri_t = setNames(dat_gryndleri_t$SE, rownames(dat_gryndleri_t))

# K-test
set.seed(123)
K_gryndleri_t <-phylosig(p.tree, response_gryndleri_t, se=se_gryndleri_t, method="K", test=TRUE)
#print(K_gryndleri_t); plot(K_gryndleri_t)
# Pagel's Lambda
set.seed(123)
lambda_gryndleri_t <-phylosig(p.tree, response_gryndleri_t, se=se_gryndleri_t, method="lambda", test=TRUE)
#print(lambda_gryndleri_t); plot(lambda_gryndleri_t)



############## H_hepaticicola_1
dat_hep1_t = dat_t %>% subset(dat_t$fungal_sp=="H_hepaticicola_1") %>%
  remove_rownames %>% column_to_rownames(var="plant_sp")

response_hep1_t = setNames(dat_hep1_t$response, rownames(dat_hep1_t))
se_hep1_t = setNames(dat_hep1_t$SE, rownames(dat_hep1_t))

# K-test
set.seed(123)
K_hep1_t <-phylosig(p.tree, response_hep1_t, se=se_hep1_t, method="K", test=TRUE)
#print(K_hep1_t); plot(K_hep1_t)
# Pagel's Lambda
set.seed(123)
lambda_hep1_t <-phylosig(p.tree, response_hep1_t, se=se_hep1_t, method="lambda", test=TRUE)
#print(lambda_hep1_t); plot(lambda_hep1_t)



############## H_hepaticicola_2
```

```
dat_hep2_t = dat_t %>% subset(dat_t$fungal_sp=="H_hepaticicola_2") %>%
  remove_rownames %>% column_to_rownames(var="plant_sp")

response_hep2_t = setNames(dat_hep2_t$response, rownames(dat_hep2_t))
se_hep2_t = setNames(dat_hep2_t$SE, rownames(dat_hep2_t))

# K-test
set.seed(123)
K_hep2_t <-phylosig(p.tree, response_hep2_t, se=se_hep2_t, method="K", test=TRUE)
#print(K_hep2_t); plot(K_hep2_t)
# Pagel's Lambda
set.seed(123)
lambda_hep2_t <-phylosig(p.tree, response_hep2_t, se=se_hep2_t, method="lambda", test=TRUE)
#print(lambda_hep2_t); plot(lambda_hep2_t)



############## H_variabilis
dat_variabilis_t = dat_t %>% subset(dat_t$fungal_sp=="H_variabilis") %>%
  remove_rownames %>% column_to_rownames(var="plant_sp")

response_variabilis_t = setNames(dat_variabilis_t$response, rownames(dat_variabilis_t))
se_variabilis_t = setNames(dat_variabilis_t$SE, rownames(dat_variabilis_t))

# K-test
set.seed(123)
K_variabilis_t <-phylosig(p.tree, response_variabilis_t, se=se_variabilis_t, method="K", test=TRUE)
#print(K_variabilis_t); plot(K_variabilis_t)
# Pagel's Lambda
set.seed(123)
lambda_variabilis_t <-phylosig(p.tree, response_variabilis_t, se=se_variabilis_t, method="lambda", test=TRUE)
#print(lambda_variabilis_t); plot(lambda_variabilis_t)



############## K_argillacea
dat_argillacea_t = dat_t %>% subset(dat_t$fungal_sp=="K_argillacea") %>%
```

```
  remove_rownames %>% column_to_rownames(var="plant_sp")

response_argillacea_t = setNames(dat_argillacea_t$response, rownames(dat_argillacea_t))
se_argillacea_t = setNames(dat_argillacea_t$SE, rownames(dat_argillacea_t))

# K-test
set.seed(123)
K_argillacea_t <-phylosig(p.tree, response_argillacea_t, se=se_argillacea_t, method="K", test=TRUE)
#print(K_argillacea_t); plot(K_argillacea_t)
# Pagel's Lambda
set.seed(123)
lambda_argillacea_t <-phylosig(p.tree, response_argillacea_t, se=se_argillacea_t, method="lambda", test=TRUE)
#print(lambda_argillacea_t); plot(lambda_argillacea_t)


############## O_maius
dat_maius_t = dat_t %>% subset(dat_t$fungal_sp=="O_maius") %>%
  remove_rownames %>% column_to_rownames(var="plant_sp")

response_maius_t = setNames(dat_maius_t$response, rownames(dat_maius_t))
se_maius_t = setNames(dat_maius_t$SE, rownames(dat_maius_t))

# K-test
set.seed(123)
K_maius_t <-phylosig(p.tree, response_maius_t, se=se_maius_t, method="K", test=TRUE)
#print(K_maius_t); plot(K_maius_t)
# Pagel's Lambda
set.seed(123)
lambda_maius_t <-phylosig(p.tree, response_maius_t, se=se_maius_t, method="lambda", test=TRUE)
#print(lambda_maius_t); plot(lambda_maius_t)


############## Serendipitaceae_sp
dat_Serendipitaceae_t = dat_t %>% subset(dat_t$fungal_sp=="Serendipitaceae_sp") %>%
  remove_rownames %>% column_to_rownames(var="plant_sp")
```

```
response_Serendipitaceae_t = setNames(dat_Serendipitaceae_t$response, rownames(dat_Serendipitaceae_t))

se_Serendipitaceae_t = setNames(dat_Serendipitaceae_t$SE, rownames(dat_Serendipitaceae_t))


# K-test

set.seed(123)

K_Serendipitaceae_t <-phylosig(p.tree, response_Serendipitaceae_t, se=se_Serendipitaceae_t, method="K", test=TRUE)

#print(K_Serendipitaceae_t); plot(K_Serendipitaceae_t)

# Pagel's Lambda

set.seed(123)

lambda_Serendipitaceae_t <-phylosig(p.tree, response_Serendipitaceae_t, se=se_Serendipitaceae_t, method="lambda", test=TRUE)

#print(lambda_Serendipitaceae_t); plot(lambda_Serendipitaceae_t)




### DATAFRAME WITH RESULTS FROM K AND LAMBDA TESTS FOR ALL FUNGI

column_names = c("K_test", "K_p_value", "Lambda_test", "Lambda_p_value")

H_bicolor_t = c(K_bicolor_t$K, K_bicolor_t$P, lambda_bicolor_t$lambda, lambda_bicolor_t$P)

H_gryndleri_t = c(K_gryndleri_t$K, K_gryndleri_t$P, lambda_gryndleri_t$lambda, lambda_gryndleri_t$P)

H_hepaticicola_1_t = c(K_hep1_t$K, K_hep1_t$P, lambda_hep1_t$lambda, lambda_hep1_t$P)

H_hepaticicola_2_t = c(K_hep2_t$K, K_hep2_t$P, lambda_hep2_t$lambda, lambda_hep2_t$P)

H_variabilis_t = c(K_variabilis_t$K, K_variabilis_t$P, lambda_variabilis_t$lambda, lambda_variabilis_t$P)

K_argillacea_t = c(K_argillacea_t$K, K_argillacea_t$P, lambda_argillacea_t$lambda, lambda_argillacea_t$P)

O_maius_t = c(K_maius_t$K, K_maius_t$P, lambda_maius_t$lambda, lambda_maius_t$P)

Serendipitaceae_sp_t = c(K_Serendipitaceae_t$K, K_Serendipitaceae_t$P, lambda_Serendipitaceae_t$lambda, lambda_Serendipitaceae_t$P)


df_t <- as.data.frame(rbind(H_bicolor_t, H_gryndleri_t, H_hepaticicola_1_t,

                H_hepaticicola_2_t, H_variabilis_t, K_argillacea_t,

                O_maius_t, Serendipitaceae_sp_t))

colnames(df_t)=column_names

df_t

write.csv2(df_t, "C:/Users/Dpao/Desktop/Master's Biology/Master Thesis/#2 semester project/Ericoid Project/phylogenetic_analysis_total.csv", row.names=TRUE)
```
